# A Method for Finding Metabolic Pathways Using Atomic Group Tracking

**DOI:** 10.1371/journal.pone.0168725

**Published:** 2017-01-09

**Authors:** Yiran Huang, Cheng Zhong, Hai Xiang Lin, Jianyi Wang

**Affiliations:** 1 School of Computer Science and Engineering, South China University of Technology, Guangzhou, China; 2 School of Computer, Electronics and Information, Guangxi University, Nanning, China; 3 Faculty of Electrical Engineering, Mathematics and Computer Science, Delft University of Technology, Delft, The Netherlands; 4 School of Chemistry and Chemical Engineering, Guangxi University, Nanning, China; Universita degli Studi di Catania, ITALY

## Abstract

A fundamental computational problem in metabolic engineering is to find pathways between compounds. Pathfinding methods using atom tracking have been widely used to find biochemically relevant pathways. However, these methods require the user to define the atoms to be tracked. This may lead to failing to predict the pathways that do not conserve the user-defined atoms. In this work, we propose a pathfinding method called AGPathFinder to find biochemically relevant metabolic pathways between two given compounds. In AGPathFinder, we find alternative pathways by tracking the movement of atomic groups through metabolic networks and use combined information of reaction thermodynamics and compound similarity to guide the search towards more feasible pathways and better performance. The experimental results show that atomic group tracking enables our method to find pathways without the need of defining the atoms to be tracked, avoid hub metabolites, and obtain biochemically meaningful pathways. Our results also demonstrate that atomic group tracking, when incorporated with combined information of reaction thermodynamics and compound similarity, improves the quality of the found pathways. In most cases, the average compound inclusion accuracy and reaction inclusion accuracy for the top resulting pathways of our method are around 0.90 and 0.70, respectively, which are better than those of the existing methods. Additionally, AGPathFinder provides the information of thermodynamic feasibility and compound similarity for the resulting pathways.

## Introduction

Finding and analyzing metabolic pathways that may span multiple organisms helps biologists to understand the metabolism, reconstruct metabolic network and discover candidate pathways for synthesis of useful biomolecules [1, 2]. The quantity and quality of metabolic data has greatly increased in the last decades [2], for instance, the metabolic databases KEGG (Kyoto Encyclopedia of Genes and Genomes) [3] and MetaCyc [4] had an exponential growth. Research on metabolic pathways on this vast quantity of metabolic data requires new computational methods in order to find and analyze biochemically relevant metabolic pathways [2]. Such computational methods can be a powerful means in discovering novel or alternative metabolic pathways that could not have been found manually [2]. Therefore, it is important to utilize novel computation methods to search and analyze alternative metabolic pathways in genome-scale database.

The efforts on studying metabolic pathways can be divided into two complementary types [5], namely stoichiometric methods and graph-based pathfinding methods. Stoichiometric methods build stoichiometry-balanced optimization models based on integer linear programming (ILP) to search for the metabolic pathway that transforms a source metabolite to a target metabolite with high yield. The stoichiometric methods are well-defined in mathematics and enable biotechnological analysis of pathways to increase the yield of important metabolites [6]. A number of the stoichiometric methods, such as CFP [7–9], PathTracer [10], OptStrain [11], OptStoic [12], NCGA [13], and RetroPath [14], has been proposed.

Graph-based pathfinding methods find possible metabolic pathways converting a given start compound to a given target compound through the connectivity of the reactions and the compounds in the metabolic networks. Some commonly used graph-based methods search metabolic pathways based on machine learning [15, 16], evolutionary algorithms [17, 18], tailored heuristic search strategy [5, 19, 20], retrosynthetic model [21–24], minimized pathway switching [25], and subgraph extraction technique [26]. Graph-based pathfinding methods complement stoichiometric methods as they focus on different aspects of modeling and understanding metabolism [2, 27–29]. Most stoichiometric methods search the pathways that obey the pseudo steady-state constraint, and therefore require assigning the internal and external metabolites [2]. This may lead to failing to find those feasible biochemical pathways that do not obey the pseudo steady-state constraint. Moreover, how to accurately assign the external metabolites which are excluded from the pseudo steady-state constraint remains a challenge [2, 27, 30]. However, both types of methods are important ways for searching and analyzing metabolic pathways [2].

A significant feature of previous graph-based pathfinding methods is that these methods select reactions and compounds based on the connectivity. However, in most cases, chemical reactions usually contain cofactors and hub metabolites such as ATP, NAD, H_2_O and H^+^ [31]. Such highly connected hub metabolites often occur in the shortest paths, and the shortest path between two compounds in metabolic networks is not always a biochemically meaningful pathway [32–34]. A possible solution to overcome the problem of hub metabolites is to remove hub metabolites from the metabolic network [19, 35, 36]. But this solution requires the user to have specialized knowledge and experience and to manually curate the networks. Moreover, if hub metabolites are removed, it is impossible to find the pathways synthetizing these compounds. Some methods were proposed to solve this problem by adding weights based on the degree of the nodes [37–39] or using structural similarity between compounds [40, 41] to guide the search of pathways. However, this does not completely avoid spurious connections occurring in the found pathways.

By providing a specific mapping from the atom in the input compounds to the atom in the output compounds of a reaction, atom mapping data offers a systematic way for understanding biochemical reactions [2]. In the past few years, the quantity and quality of atom mapping information have been steadily increasing, with one of the main sources being the KEGG RPAIR database [3, 42]. Recently, people use atom mapping data to avoid spurious connections when searching pathways [32, 43]. Based on the observation that the same atom-mapping pattern between two compounds often appears in multiple reactions [32], some researchers [44, 45] utilized atom mapping data to find metabolic pathways by allowing only connections through reactions where at least one atom is being transferred from the input to the output compounds. However, the pathways that conserve the atoms from start to target compounds will be more biochemically relevant [46]. Some pathfinding methods using atom tracking have been developed to find such pathways. ReTrace [30] and LPAT [2, 46] use atom mapping information from the KEGG RPAIR database to search metabolic pathways that conserve at least a given number of atoms from the start to the target compounds, their experimental results showed that atom tracking significantly improves the performance of metabolic pathfinding. Different from the methods using atom mapping data from databases, MetaRoute [1, 6] automatically computes atom mapping rules based on enzyme EC numbers and compound SMILES, and uses the computed atom mapping data to avoid finding pathways that lose all conserved atoms from the start compound. MetaRoute correctly returned textbook-like routes, e.g. it recovered a major part of glycolysis. RouteSearch [47] uses a branch-and-bound algorithm to compute the optimal metabolic pathways, where optimality is based on the number of reactions used, the provenance of the reactions and the atoms conserved by the route from the start to target compounds. RouteSearch successfully found the known pathways with a larger efficiency than previous methods.

Heath *et al*. [46] pointed out that atom tracking is a very important feature in finding meaningful metabolic pathways since it essentially excludes spurious connections and reactions that do not correspond to useful or real biochemical pathways. However, in order to track the movements of target atoms, the pathfinding methods using atom tracking require the user to define the specific atoms to be tracked in advance. This may lead to failing to predict the pathways that do not conserve the user-defined atoms.

A synopsis on the pathfinding tools for metabolic pathway is listed in Table 1.

**Table 1:** A synopsis on the pathfinding tools for metabolic pathway.

Name	Description	Reference
CFP	stoichiometric method based on mixed-integer linear programming	[7–9]
PathTracer	stoichiometric method using flux analysis of metabolic pathways	[10]
OptStrain	stoichiometric method based on mixed-integer linear programming	[11]
OptStoic	stoichiometric method based on mixed-integer linear programming	[12]
NCGA	stoichiometric method combining newton method and genetic algorithm	[13]
RetroPath	stoichiometric method using flux analysis of metabolic pathways	[14]
Pathways Tool	graph-based method based on machine learning	[15]
EAMP	graph-based method based on evolutionary algorithms	[17]
EvoMS	graph-based method based on evolutionary algorithms	[18]
FogLight	graph-based method based on tailored heuristic search strategy	[5]
Tinker	graph-based method based on tailored heuristic search strategy	[19]
PathMiner	graph-based method based on tailored heuristic search strategy	[20]
FindPath	graph-based method based on retrosynthetic model	[23]
GEM-Path	graph-based method based on retrosynthetic model	[21]
CMPF	graph-based method based on minimized pathway switching	[25]
NeAT	graph-based method based on subgraph extraction technique	[26]
Rahnuma	graph-based method based on hypergraph search	[48]
MRSD	graph-based method based on the weighted compound transform diagraph search	[38]
SimIndex and SimZyme	graph-based method based on compound similarity	[40]
PHT	graph-based method based on compound similarity	[41]
ReTrace	graph-based method using atom tracking	[30]
LPAT	graph-based method using atom tracking	[2, 46]
MetaRoute	graph-based method using atom tracking	[1, 6]
RouteSearch	graph-based method using atom tracking	[47]

In this article, we present a pathfinding method called AGPathFinder to find biochemically relevant metabolic pathways. Our method differs from the atom tracking methods by tracking the movement of atomic groups through metabolic networks and implementing a shortest-path-based algorithm that uses combined information of reaction thermodynamics and compound similarity both to direct its search for the pathways between two desired compounds and to rank the resulting pathways. Atomic group tracking enables our method to avoid hub metabolites and search pathways without requiring the user to define the atoms to be tracked. Meanwhile, atomic group tracking, when combined with the information of reaction thermodynamics and compound similarity, can further improve the quality of the found pathways. The experimental results show that AGPathFinder is capable of finding both known pathways and thermodynamically feasible alternative pathways. Compared with other previous methods, our method finds alternative pathways with a higher accuracy and lower error in genome-scale database.

The remaining of the article is organized as follows. Section “Method” introduces the weighted atomic group transfer graph and presents our method AGPathFinder. Section “Results” describes the experimental setup and study cases, compares the results with other existing methods. Section “Discussion and Conclusion” concludes the article.

## Method

### Atomic group transfer graph

Zhou and Nakhleh argued that two atoms in a compound are considered to be in the same atomic group if they are linked by covalent bond(s) that does not break during the chemical reactions [45]. Accordingly, an atomic group is a group of atoms transferred between a substrate and a product in the reaction, where the covalent bonds between the atoms in the group do not break during the reaction. The size of an atomic group is determined by the number of atoms in the group. Due to the fact that any atom could be a member of an atomic group, we can use atomic groups, instead of specific atoms, as the targets and track the movements of atomic groups through metabolic networks to find biochemically relevant pathways. This will not require the user to define the specific atoms to be tracked and allows the user to find pathways without even knowing the atoms of the compound. Moreover, since the amount of chemical content is measured in terms of the number of functionally independent atomic groups instead of the absolute number of non-hydrogen atoms [45], the pathways that conserve at least one atomic group from the start to target compounds will be more biochemically relevant. During the pathway inference, a conserved atomic group in the pathway is a group of atoms transferred from start compound to current compound, where the covalent bonds between the atoms in the group do not break during the reactions in the pathway.

In this work, we use the atom mapping data of reactions in the KEGG RPAIR database to compute the atomic group transferred between reactants and products. Each KEGG RPAIR entry contains the structural information for each compound, an alignment mapping atoms between the two compounds, and a list of associated reactions [42, 46]. The KEGG RPAIR data do not contain typical molecular symmetry information. If a compound is known to be symmetric, a new atom mapping entry can be generated to illustrate symmetry of the molecules [46]. When we need to process symmetry of the molecules, we only add those atom mapping entries explicitly appeared in the KEGG RPAIR data.

A chemical compound can be represented as an attributed relational graph *K*, whose set of nodes *V*(*K*) correspond to atoms and set of edges *E*(*K*) correspond to chemical bonds [49]. A node *v*∈*V*(*K*) refers to an atom and an edge *e*∈*E*(*K*) refers to a chemical bond. Given two compounds *G* and *H*, *u*,*v*∈*V*(*G*), *m*,*n*∈*V*(*H*), (*u*,*v*)∈*E*(*G*), (*m*,*n*)∈*E*(*H*), *R* is a chemical reaction between *G* and *H*, and *f* is a reaction atom mapping of *R* in the RPAIR database: *V*(*G*)→*V*(*H*). If *f*(*u*) = *m* and *f*(*v*) = *n*, then (*u*,*v*)→(*m*,*n*) is an edge mapping from *G* to *H*.

**Fig 1:**
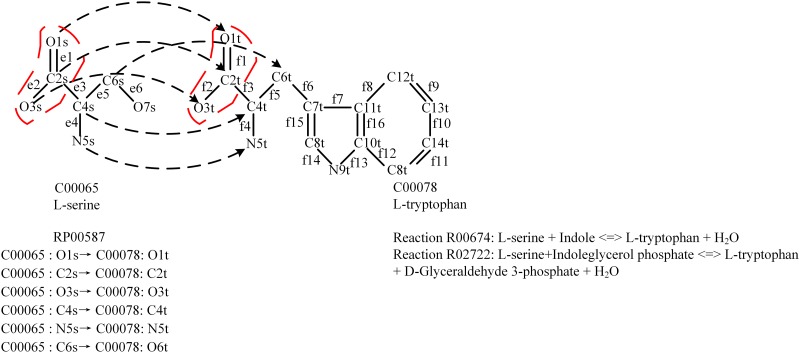
Conserved atomic group transfer. Conserved atomic group transfer in chemical reactions R02722 and R00674 in KEGG RPAIR database. R02722: L-serine+Indoleglycerol phosphate < = > L-tryptophan + D-Glyceraldehyde 3-phosphate + H_2_O. R00674: L-serine + Indole < = > L-tryptophan + H_2_O. The arrows denote mapping of atoms from C00065 to C00078 via R02722 and R00674. The partition encircled with dotted line is the conserved atomic group transferred from the start compound during the pathway inference. Atom mapping entry RP00587 contains reactions R00674 and R02722. Hydrogens and their associated bonds are not shown.

In this article, reactions and compounds are represented by their KEGG identifiers. Fig 1 describes a conserved atomic group transferred in chemical reactions R02722 and R00674 during the pathway inference, where compound C00065 is composed of the atom set *G*_*a*_ = {O1s, C2s, O3s, C4s, N5s,C6s,O7s} and the bond set *G*_*b*_ = {e1,e2,e3,e4,e5,e6}, compound C00078 is composed of the atom set *H*_*a*_ = {O1t, C2t, O3t, C4t, N5t,C6t, C7t,C8t, N9t,C10t, C11t, C12t, C13t,C14t,C15t} and the bond set *H*_*b*_ = {f1,f2,f3,f4, f5,f6,f7, f8,f9, f10, f11, f12,f13, f14, f15,f16}.

From Fig 1 we can obtain atom mapping *f*: {C00065:O1s→C00078:O1t, C00065:C2s→C00078:C2t, C00065:O3s→C00078:O3t, C00065:C4s→C00078:C4t, C00065:N5s→C00078:N5t, C00065:C6s→C00078:C6t} between C00065 and C00078 from the atom mapping entry RP00587 in KEGG RPAIR database. Based on atom mapping *f*, we obtain the corresponding edge mapping *h*:{e1→f1, e2→f2, e3→f3, e4→f4, e5→f5}. For simplicity, we assume that the partition circled with dotted line in C00065 is a conserved atomic group transferred from the start compound to C00065 during the pathway inference, the atoms in this atomic group are {O1s,C2s,O3s}⊆*V*(C00065), and the bonds between these atoms are {e1,e2}⊆*E*(C00065). Sub-structure of C00078 (the partition encircled with dotted line in C00078) with atoms {O1t,C2t,O3t}⊆*V*(C00078) and bonds {f1,f2}⊆*E* (C00078) forms a conserved atomic group that is transferred from C00065 to C00078 through reactions R02722 and R00674.

An atomic group transfer graph can be represented as a directed metabolite graph, whose nodes are compounds and edges represent reactions linking an input compound and an output compound. Each edge contains at least one atomic group transferred from the input compound to the output compound.

**Fig 2:**
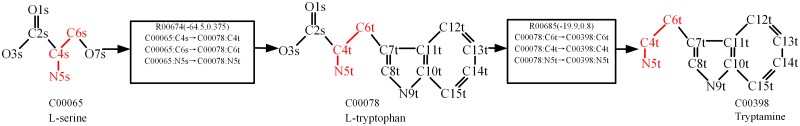
Illustration of an atomic group transfer graph. The atomic group transfer graph contains three compounds and two reactions, where reaction R00674 links input compound C00065 and output compound C00078, reaction R00685 links input compound C00078 and output compound C00398, the data in parentheses denote the Gibbs free energy and compound similarity respectively, hydrogens and their associated bonds are not shown.

Fig 2 shows an instance of atomic group transfer graph, where three atoms (C4s, C6s, N5s) of compound C00065 and the bonds between these atoms form an atomic group that is transferred from C00065 to C00398 through R00674 and R00685.

During the pathway inference, we use the reactions and compounds that contain conserved atomic groups to construct an atomic group transfer graph from start to target compounds. Then we search biochemically relevant pathways that transfer the conserved atomic groups from start compound to target compound in the graph.

### Weighting schemes

In addition to using atomic group tracking to find biochemically relevant metabolic pathways, we also introduce the weighting schemes based on the associated context-specific knowledge including reaction thermodynamics and structural similarity between reactant and product. We can use such weighting schemes to guide the search process towards more feasible pathways and better performance, and to find meaningful pathways even without the option of tracking atomic groups. In our weighting schemes, each edge in an atomic group transfer graph will be assigned with a weight that reflects the impact of the reaction thermodynamics and the structural similarity between reactant and product on the alternative pathways.

### Thermodynamic information on reactions

Gibbs free energy is usually used to determine whether a reaction or metabolic pathway is thermodynamic feasible [50]. We use $\Delta {G^{\prime }}_{r}$ to denote the Gibbs free energy change of reactions in KEGG RPAIR database. The corresponding values of $\Delta {G^{\prime }}_{r}$ of the reactions are obtained from the literature [50], which are downloaded from “Group Contribution Data” in the table “Reaction Energies” at http://equilibrator1.milolab.webfactional.com/download. The value of $\Delta {G^{\prime }}_{r}$ of a reaction is an essential part of the edge weight and it also provides a means of ranking the results. For example, from Fig 2 we can see that R00674 and R00685 are represented as two edges of an atomic transfer graph. The values of $\Delta {G^{\prime }}_{r}$ of R00674 and R00685 are -64.5 and -19.9. These values of $\Delta {G^{\prime }}_{r}$ are used as a part of the weights for R00674 and R00685 (for more details see section “Weight computation”). In the process of finding candidate pathways in atomic group transfer graph, we can calculate the sum of the weights of all edges of each pathway from the start to target compound, and rank the pathway by the sum (for more details see section “Constructing atomic group transfer graph and finding candidate pathways”). User can analyze thermodynamic feasibility of each pathway by the values of $\Delta {G^{\prime }}_{r}$ of reactions. In this article, the values of $\Delta {G^{\prime }}_{r}$ of reactions under the conditions of pH = 7.0, ionic strength = 0.1, and T = 298.15K are downloaded from “Group Contribution Data” in the table “Reaction Energies” at http://equilibrator1.milolab.webfactional.com/download.

### Compound similarity

In addition to reaction thermodynamics, the structural similarity between two compounds is widely used to measure the diversity of the chemical space and analyze the metabolic networks [20, 41, 51]. For example, the SMSD tool [51] has been applied to compute the structural similarity between two compounds. In this article, we use SMSD to compute the similarity scores between the input compounds and output compounds in all reactions in a pathway. This similarity score is used as a part of the edge weight to guide the search process, which will be further described in the section “Weight computation”.

### Weight computation

AGPathFinder uses the combined information of structural similarity between compounds and reaction thermodynamics to weight the edges, and AGPathFinder moves to the edges that are more thermodynamically favorable and/or link with more structurally similar nodes. Given an atomic group transfer graph *G*_*ag*_ = (*V*_*ag*_, *E*_*ag*_) with node set *V*_*ag*_ and edge set *E*_*ag*_, nodes *v*_*i*_ and *v*_*j*_∈*V*_*ag*_ denote two compounds in *G*_*ag*_. An edge *e*_*ij*_∈*E*_*ag*_ linking *v*_*i*_ and *v*_*j*_ represents a reaction *r*_*ij*_, where reaction *r*_*ij*_ contains the atomic group transferred between compounds *v*_*i*_ and *v*_*j*_. We represent the compound similarity between *v*_*i*_ and *v*_*j*_ by *sim*(*v*_*i*_,*v*_*j*_) and the $\Delta {G^{\prime }}_{r}$ of reaction *r*_*ij*_ by *fe*(*r*_*ij*_), and compute the weight *W*_*ij*_ of edge *e*_*ij*_ as follows:

$$W_{ij}=\alpha (1-sim(v_{i},v_{j}))+(1-\alpha )(3200+fe(r_{ij}))/10000$$ (1)

where *α* is a parameter adjusting relative weights of compound similarity and Gibbs free energy, and the constants 3200 and 10000 are used to normalize the value of *fe*(*r*_*ij*_). In Eq (1), the value of *sim*(*v*_*i*_,*v*_*j*_) is between 0 and 1, and the value of *fe*(*r*_*ij*_) downloaded from the table “Reaction Energies” at http://equilibrator1.milolab.webfactional.com/download is between 10194.7 and -2233.7. That is to say, the difference between *sim*(*v*_*i*_,*v*_*j*_) and *fe*(*r*_*ij*_) is very large. The normalization of *fe*(*r*_*ij*_) in Eq (1) adjusts the value of *fe*(*r*_*ij*_) to the range [0.09663, 1.33947] and brings the values of *sim*(*v*_*i*_,*v*_*j*_) and *fe*(*r*_*ij*_) into alignment.

In Fig 2, according to Eq (1), when *α* = 0.5, the values of weight *W*_*ij*_ for reactions R00674 and R00685 are 0.469275 and 0.259005 respectively; when *α* = 1, the weight *W*_*ij*_ only depends on the similarity between *v*_*i*_ and *v*_*j*_, and the values of *W*_*ij*_ for R00674 and R00685 are 0.625 and 0.2 respectively; when *α* = 0, the weight *W*_*ij*_ only depends on $\Delta {G^{\prime }}_{r}$ of reaction *r*_*ij*_, and the values of *W*_*ij*_ for R00674 and R00685 become 0.31355 and 0.31801 respectively.

### Constructing atomic group transfer graph and finding candidate pathways

To construct an atomic group transfer graph from the start compound to the target compound, we need to compute the information for the atomic group transferred from substrate to product through reaction. Given substrate *G* and product *H* in reaction *R* and a user-specified size of atomic group, the following CAGM algorithm finds all conserved atomic groups of the user-specified size or larger transferred from *G* to *H* through reaction *R* [Algorithm 1].

**Algorithm 1: CAGM**

**Input:** substrate *G* in reaction *R*, product *H* in reaction *R*, conserved atomic group set *R*_*g*_ of *G* from start compound, edge mapping *h* for reaction *R*, user-specified size *L* of atomic group;

**Output:** conserved atomic group set *S* of *H*; subgraph *M* of *H*;

1. *S*←*Φ*;

2. **for each** edge *e*(*m*_1_, *m*_2_)∈*E*(*R*_*g*_) where *m*_1_, *m*_2_∈*V*(*R*_*g*_) **do**

3.  **if** *h*(*e*) = *e*′ where *e*′(*m*_1_′,*m*_2_′)∈*E*(*H*) and *m*_1_′, *m*_2_′∈*V*(*H*) **then**

4.   *V*(*M*)←{*m*_1_′,*m*_2_′},where *V*(*M*)⊆*V*(*H*);

5.   *E*(*M*)←(*m*_1_′,*m*_2_′), where *E*(*M*)⊆*E*(*H*);

 end if

end for

6. **for each** unvisited node *m* in *M* **do**

7.  Find the connected component *MC* containing *m* in *M* by the depth-first search algorithm;

8.  Mark each node in *MC* as visited;

9.  **if** the number of nodes in *MC*≥ *L* **then**

10.   *S*←*S*∪{*MC*}, where *MC*⊆*M*;

 end if

end for

11. **Return** *S*.

Initially, if *G* is a start compound, then *G* is the only molecular in *R*_*g*_. Let *h* be an edge mapping from *G* to *H*. At the beginning, CAGM finds all mapping edges from *R*_*g*_ to *H* by using *h*, and then uses these edges to construct subgraph *M* of *H* (lines 2–5). For each unvisited node *m* in *M*, the connected component *MC* containing *m* in *M* is determined by the depth-first search algorithm (lines 6–7), and each node in *MC* is then marked as visited (line 8). If the number of nodes in *MC*≥*L*, then *MC* is added to *S* (lines 9–10). Repeat this procedure until all nodes in *M* are visited.

In the following we use an example to explain the algorithm in finding the conserved atomic group transferred from substrate to product through reaction R02722 in Fig 1.

Example 2.1: Compound C00065 is the substrate *G* and compound C00078 is the product *H*. Let *L* = 2. The partition encircled with dotted line in C00065 is the conserved atomic group transferred from a start compound to C00065. This conserved atomic group constructs the conserved atomic group set *R*_*g*_ of C00065. At the beginning, we find all mappings of the edges {e1,e2} of *R*_*g*_ in C00078 by edge mapping *h*:{e1→f1, e2→f2, e3→f3, e4→f4, e5→f5}, and the resulting edge mappings are {e1→f1, e2→f2}. We then use f1 and f2 to construct a subgraph *M* of C00078. For each unvisited node *m*∈{O1t,C2t,O3t} in *M*, we find the connected component *MC* containing *m* in *M* by a depth-first search algorithm, and mark each node of *MC* as visited. From Fig 1, we can see that the atom set {O1t,C2t,O3t}⊆*V*(C00078) and the bond set {f1,f2}⊆*E*(C00078) form the *MC*. It is obvious that the number of nodes in *MC*≥2, thus *MC* is added to *S*, the algorithm terminates here since all nodes in *M* are visited.

The algorithm CAGM finds potential atomic groups transferred from substrates to products through reaction. Given start compound *Sm* and target compound *Tm*, the following CAGTG algorithm creates a weighted atomic group transfer graph *G*_*ag*_ between *Sm* and *Tm*, and finds the top *k*-shortest paths *C*_*p*_ with the smallest weight from *Sm* to *Tm* in *G*_*ag*_ [Algorithm 2].

**Algorithm 2: CAGTG**

**Input:** start compound *Sm*, target compound *Tm*, conserved atomic group set *R*_*g*_ from start compound, Boolean vector *ψ*(*Sc*,*Td*), where *Sc* denotes compound similarity, *Td* denotes thermodynamic feasibility;

**Output:** weighted atomic group transfer graph *G*_*ag*_ between *Sm* and *Tm*, top *k*-shortest paths *C*_*p*_ with the smallest weight from *Sm* to *Tm* in *G*_*ag*_;

1. Mark *Sm* as visited;

2. Add *Sm* to *G*_*ag*_;

3. Queue *Q*←*Sm*;

4. **While** queue *Q* is not empty **do**

5.  *v*_*i*_←pop(*Q*);

6.  **If** *v*_*i*_ is not *Tm* **then**

7.   **for each** unvisited node *v*_*j*_ adjoining to *v*_*i*_ **do**

8.    Compute the conserved atomic group set *S* transferred from *v*_*i*_ to *v*_*j*_ by using algorithm CAGM;

9.    Mark *v*_*j*_ as visited;

10.    **If** *S* is not empty **then**

11.     Compute the weight of edge (*v*_*i*_, *v*_*j*_) by *W*_*ij*_ = *α*(1-*sim*(*v*_*i*_,*v*_*j*_))+(1-*α*)(3200+*fe*(*r*_*ij*_))/10000 with Boolean vector *ψ*(*Sc*,*Td*);

12.     Concatenate *v*_*j*_ to *v*_*i*_ in *G*_*ag*_, add *v*_*j*_ and edge (*v*_*i*_, *v*_*j*_) to *G*_*ag*_;

13.     *Q*←{*v*_*j*_}∪*Q*;

14.     Replace the conserved atomic group set *R*_*g*_ of *v*_*j*_ with *S*.

   end if

  end for

 end if

end while

15. Determine the top *k*-shortest paths *C*_*p*_ with the smallest weight between *Sm* and *Tm* in *G*_*ag*_.

16. **Return** *C*_*p*_

In an iterative manner, algorithm CAGTG removes node *v*_*i*_ from queue *Q* (lines 4–5), where *Q* is the set of candidate nodes and these candidate nodes are used to create *G*_*ag*_. If node *v*_*i*_ is not the target compound (line 6), for each unvisited node *v*_*j*_ adjoining to *v*_*i*_, CAGTG executes algorithm CAGM to compute the conserved atomic groups transferred from *v*_*i*_ to *v*_*j*_ (lines 7–8), and mark node *v*_*j*_ as visited (line 9). If *S* is not empty (line 10), CAGTG computes the weight of edge (*v*_*i*_, *v*_*j*_) by (Eq (1) in Section “Weight computation”)according to the value of *ψ*(*Sc*,*Td*) (lines 10–11), add node *v*_*i*_ and edge (*v*_*i*_, *v*_*j*_) to *G*_*ag*_ (line 12), put node *v*_*j*_ in *Q* (line 13), and replace the conserved atomic group set *R*_*g*_ of *v*_*j*_ with *S* (line 14). CAGTG repeats this procedure until *Q* is empty. When *Q* is empty, the atomic group transfer graph between *Sm* and *Tm* has been created. Finally, CAGTG has found the top *k*-shortest paths *C*_*p*_ with smallest weight from *Sm* to *Tm* in *G*_*ag*_ as candidate paths (lines 15–16).

Our algorithm CAGTG provides two user-defined searching parameters *Sc* and *Td*, which allow the user to manipulate the parameter *α* in Eq (1) to guide the search for specific pathways of interest. For example, when we want to find the pathways that consist of reactions with low $\Delta {G^{\prime }}_{r}$, we can set *ψ*(*Sc*, *Td*) = *ψ*(*false*, *true*). If *ψ*(*Sc*, *Td*) = *ψ*(*false*, *true*), AGPathFinder uses *α* = 0 and it means that the weight of edge in Eq (1) is determined by Gibbs free energy and the search will be driven by Gibbs free energy. When we want to find the pathways that consist of similar compounds, we can set *ψ*(*Sc*, *Td*) = *ψ*(*true*, *false*). If *ψ*(*Sc*, *Td*) = *ψ*(*true*, *false*), AGPathFinder uses *α* = 1and it means that the weight of edge in Eq (1) is determined by compound similarity and the search will be driven by compound similarity. When we want to find the pathways that consist of reactions with low $\Delta {G^{\prime }}_{r}$ and similar compounds, we can set *ψ*(*Sc*,*Td*) = *ψ*(*true*, *true*). If *ψ*(*Sc*,*Td*) = *ψ*(*true*, *true*), AGPathFinder uses *α* = 0.5 and it means that the weight of edge in Eq (1) is determined by compound similarity and Gibbs free energy and the search will be driven by compound similarity and Gibbs free energy.

The following example illustrates the process of creating weighted atomic group transfer graph between two compounds.

**Fig 3:**
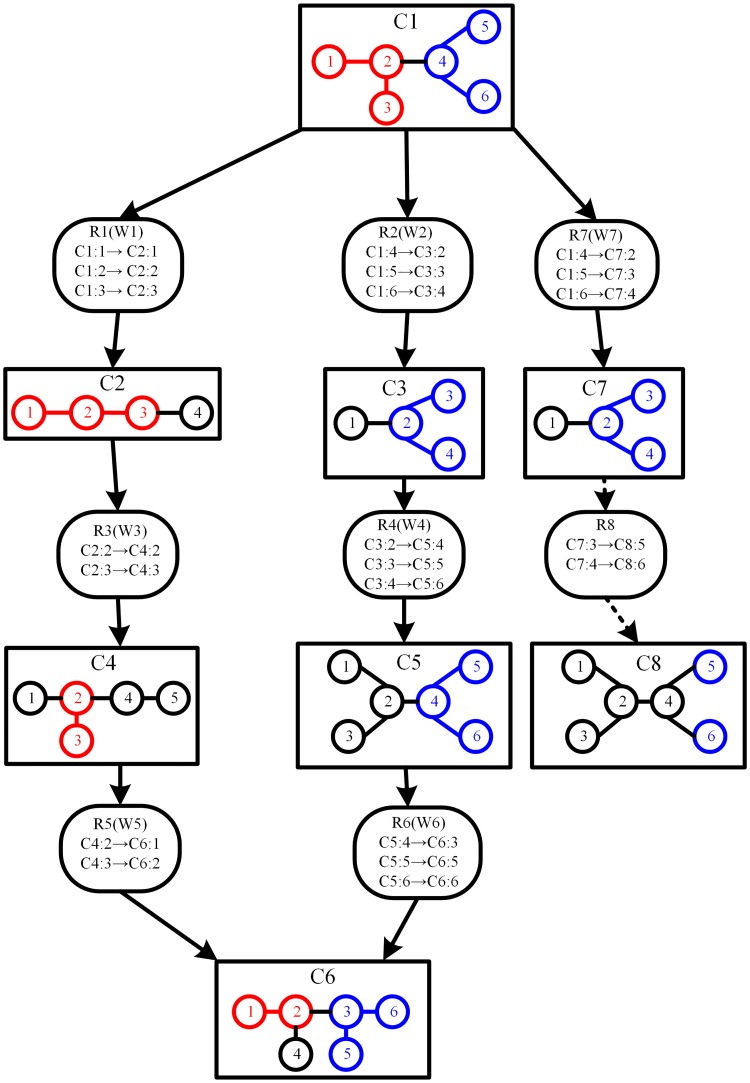
An abstract representation of weighted atomic group transfer graph *G*_*ag*_. A square rectangle represents a compound node. The atoms are represented as circles. C1, C2, C3, C4, C5, and C6 are the compound identifiers. The edges linking atoms represent chemical bonds, and the rounded rectangles represent reactions that contain the atom mappings between compounds, with the reaction identifiers being R1, R2, R3, R4, R5, R6, R7 and R8. W1, W2, W3, W4, W5, W6 and W7 are the weights of the edges R1, R2, R3, R4, R5, R6 and R7 respectively. Both red atoms and blue atoms are the conserved atoms from start compound C1. In compound C6, the group of red atoms with associated bonds and the group of blue atoms with associated bonds are two conserved atomic groups transferred from start compound C1 to target compound C6. Since the conserved atoms transferred from C7 to C8 through R8 do not form atomic group in C8, R8 and C8 are shown with arrows in dotted line to indicate that R8 and C8 do not exist in *G*_*ag*_.

Example 2.2: Fig 3 shows an abstract representation of a weighted atomic transfer graph *G*_*ag*_ between start compound C1 and target compound C6. At the beginning of algorithm CAGTG, there is only one node in *G*_*ag*_. We put C1 in queue *Q*. In an iterative manner, we remove a node from *Q* each time. The first node removed from *Q* is C1, which is not the target compound. For the unvisited nodes C2, C3 and C7 adjoining to C1, we use algorithm CAGM to compute the atomic groups transferred from C1 to C2, C3 and C7 respectively, the resulting atomic groups are S2, S3 and S7. The atomic groups S2, S3 and S7 consist of sets of atoms {1,2,3}⊆*V*(C2), {2,3,4}⊆*V*(C3), and {2,3,4}⊆*V*(C7) respectively. The associated bond sets of S2, S3, S7 are {(1,2),(2,3)}⊆*E*(C2), {(2,3),(2,4)}⊆*E*(C3) and {(2,3),(2,4)}⊆*E*(C7) respectively. We mark nodes C2, C3 and C7 as visited. Since the resulting atomic groups S2, S3 and S7 are not empty, we use Eq (1) to compute the weights W1, W2, W7 of edges R1, R2 and R7 respectively. Then we add nodes C2, C3, C7 and edges R1, R2, R7 to *G*_*ag*_, and put C2, C3, C7 in queue *Q*. We replace the conserved atomic group sets of C2, C3 and C7 with S2, S3 and S7 respectively. Next, the node to be removed from *Q* is C2 which is (again) not the target compound. For the unvisited node C4 adjoining to C2, we use CAGM to compute the conserved atomic groups transferred from C2 to C4, the resulting atomic group is S4 with the atom set {2,3}⊆*V*(C4) and the bond set {(2,3)}⊆*E*(C4). Node C4 is marked as visited. Since S4 is not empty, we compute the weight W3 of edge R3 by Eq (1). Then we add node C4 and edge R3 to *G*_*ag*_, and put C4 in queue *Q*. We replace the conserved atomic group set of C4 with S4. This procedure is repeated until *Q* is empty. When *Q* is empty, the atomic group transfer graph between C1 and C6 has been created. Now the top *k*-shortest paths can be determined from *G*_*ag*_. For instance, if *k* = 2, we determine the top 2 shortest paths with the smallest weight from C1 to C6 in *G*_*ag*_ as candidate metabolic pathways, and these 2 pathways are C1→R1→C2→R3→C4→R5→C6 and C1→R2→C3→R4→C5→R6→C6.

## Results

From the KEGG LIGAND database, we obtained 5848 compound structures and 7340 reactions which have corresponding KEGG RPAIR entries. We used the SMSD tool to compute the similarity between compounds. The atomic group transfer graph is built based on the KEGG RPAIR database. We have implemented AGPathFinder in Java.

In order to evaluate the performance of AGPathFinder, the results are compared with several available metabolic pathfinding methods using atom tracking and an available graph-based method Tinker [19]. These atom tracking methods are RouteSearch [47], LPAT [46] and ReTrace [30] which are the software available and currently maintained. Tinker [19] is a recently developed method that finds pathways based on tailored heuristic search strategy and requires excluding hub metabolites. In the experiments, we use a set of 42 known pathways (as detailed in S1 Text) that were derived from the aMAZE database [52] and were commonly used for the evaluation of pathfinding methods in the literature [46]. The five methods AGPathFinder, RouteSearch, Tinker, LPAT and ReTrace are used to compute the pathways between the start and target compounds of each of the 42 known pathways. Then we compare the computed pathways with the corresponding known pathways to evaluate the performance of the methods. Furthermore, three study cases will be carried out to learn more about the characteristics of these methods.

RouteSearch is a web-based pathfinding tool. We used RouteSearch to search pathways on Biocyc.org. We downloaded Tinker, LAPT and ReTrace from http://osslab.lifesci.warwick.ac.uk/ tinker.aspx, http://www.kavrakilab.org/atommetanet and http://www.cs.helsinki.fi/group/sysfys/software/ReTrace respectively. AGPathFinder, LPAT and ReTrace were run on the Sugon 5000A parallel computer at Guangxi University, using a single computing node with a quad-core Intel(R) Xeon(R) CPU E5620 @ 2.40GHz and 40GB RAM. The running operating system is Linux. Tinker was implemented in C# and runs on a PC with Intel(R) Pentium(R) CPU G3240 @ 3.10GHz and 8GB RAM, and the running operating system is Windows 7. When Tinker was run to search pathways, the hub metabolites listed in [19] (see also S1 Table) are excluded in advance.

### Comparing computed pathways to known pathways

For each pathway, we use measures defined previously in the literature [46] to compute the accuracy *Ac*, sensitivity *Sn* and positive prediction value *PPV* to evaluate the biochemical performance of pathways computed by AGPathFinder, RouteSearch, Tinker, LPAT and ReTrace. To describe these measures, we need to define the correct compounds in the computed pathway. The compounds in the computed pathway are called correct compounds if these compounds satisfy the following two conditions: (1) The compounds can be found in both computed and known pathways, which are called included compounds. (2) The order of the included compounds in the computed pathway is the same as the order of the included compounds in the known pathway.

Then the values of *Sn* and *PPV* are defined as follows: *Sn* = *TP*/(*TP*+*FN*) and *PPV* = *TP*/(*TP*+*FP*), where true positives (*TP*) are the correct compounds found in the computed pathway, false negatives (*FN*) are the compounds in the known pathway but not in the computed pathway, and false positives (*FP*) are the compounds not in the known pathway but in the computed pathway [46]. Because true negatives do not exist in this comparison, *Ac* = (*Sn*+*PPV*)/2 [46]. We use cross-validation [53] to estimate the error *Er* for the compounds between computed pathway and known pathway. The smaller the error *Er* is, the more similar the computed pathway and the known pathway are. We can use the error *Er* to analyze the ability of pathfinding methods in recovering known pathways. Besides *Ac*, *Sn*, *PPV* and *Er*, we also use *F*-measure *Fm* for compound to evaluate the performance of pathfinding methods, where *Fm* = (2×*PR*×*RC*)/(*PR*+*RC*), *PR* is the precision and *PR* = *PPV*, *RC* is the Recall and *RC* = *Sn*, and Recall is the proportion of positive cases [54].

In addition to measuring the performance of the computed pathway based on compound, we also measure the performance of the computed pathways based on reaction. By analogy with the definition of correct compound, we derive the definition of the correct reaction in the computed pathways. The reactions in the computed pathway are called correct reactions if these reactions satisfy the following two conditions: (1) The reactions can be found in both computed and known pathways, which are called included reactions. (2) The order of the included reactions in the computed pathway is the same as the order of the included reactions in the known pathway.

The values of the sensitivity *Sn_R* and positive prediction value *PPV_R* for reaction are defined as follows: *Sn_R* = *TP_R*/(*TP_R*+*FN_R*) and *PPV_R* = *TP_R*/(*TP_R*+*FP_R*), where true positives (*TP_R*) are the correct reactions found in the computed pathway, false negatives (*FN_R*) are the reactions in the known pathway but not in the computed pathway, and false positives (*FP_R*) are the reactions not in the known pathway but in the computed pathway. The accuracy for reaction is *Ac_R* = (*Sn_R*+*PPV_R*)/2. By analogy with *Er*, we also use cross-validation [53] to estimate the error *Er_R* for the reactions between computed pathway and known pathway. Besides *Ac_R*, *Sn_R*, *PPV_R* and *Er_R*, we also use *F*-measure *Fm_R* for reaction to evaluate the performance of pathfinding methods, where *Fm_R* = (2×*PR_R*×*RC_R*)/(*PR_R* +*RC_R*), *PR_R* is the precision and *PR_R* = *PPV_R*, *RC_R* is the Recall and *RC_R* = *Sn_R*.

### AGPathFinder versus other methods

In this section, for each pair of start and target compounds of the 42 known pathways in the test, we use AGPathFinder, RouteSearch, Tinker, LPAT and ReTrace to find the top ten pathways. These top ten pathways are then compared to the known pathways and the results are shown in Tables 2 and 3.

**Table 2:** Average accuracy, sensitivity, positive prediction value, *F*-measure and error of including specific compounds in the 42 computed pathways.

Method	Top Path					Best of top ten paths				
	*Ac*	*PPV*	*Sn*	*Fm*	*Er*	*Ac*	*PPV*	*Sn*	*Fm*	*Er*
RouteSearch	0.90	0.91	0.88	0.90	0.05	0.94	0.94	**0.94**	0.94	0.04
Tinker	0.77	0.76	0.78	0.77	0.21	0.85	0.85	0.85	0.85	0.14
LPAT	0.81	0.85	0.77	0.81	0.17	0.86	0.92	0.79	0.85	0.13
Retrace	0.68	0.67	0.70	0.68	0.27	0.87	0.90	0.84	0.87	0.07
AGPathFinder with *ψ*(*Sc*,*Td*) = *ψ*(*true*,*true*)	0.91	0.94	0.88	0.91	**0.03**	**0.95**	**0.97**	**0.94**	**0.95**	**0.01**
AGPathFinder with *ψ*(*Sc*,*Td*) = *ψ*(*true*,*false*)	**0.92**	**0.95**	**0.90**	**0.92**	0.04	0.94	0.96	0.92	0.94	**0.01**
AGPathFinder with *ψ*(*Sc*,*Td*) = *ψ*(*false*,*true*)	0.88	0.92	0.84	0.88	0.07	0.91	0.95	0.88	0.91	0.05

The best performer for the relative item is marked in bold.

**Table 3:** Average accuracy, sensitivity, positive prediction value, *F*-measure and error of including specific reactions in the 42 computed pathways.

Method	Top Path					Best of top ten paths				
	*Ac_R*	*PPV_R*	*Sn_R*	*Fm_R*	*Er_R*	*Ac_R*	*PPV_R*	*Sn_R*	*Fm_R*	*Er_R*
RouteSearch	0.64	0.64	0.63	0.64	0.20	0.77	0.77	0.76	0.76	**0.06**
Tinker	0.49	0.49	0.49	0.49	0.28	0.64	0.64	0.64	0.64	0.16
LPAT	0.55	0.55	0.56	0.55	0.20	**0.78**	**0.78**	**0.77**	0.77	**0.06**
Retrace	0.36	0.37	0.36	0.36	0.40	0.72	0.73	0.71	0.72	0.09
AGPathFinder with *ψ*(*Sc*,*Td*) = *ψ*(*true*,*true*)	**0.70**	**0.71**	**0.69**	**0.70**	**0.14**	**0.78**	**0.78**	**0.77**	**0.78**	**0.06**
AGPathFinder with *ψ*(*Sc*,*Td*) = *ψ*(*true*,*false*)	**0.70**	0.70	**0.69**	**0.70**	**0.14**	**0.78**	**0.78**	**0.77**	**0.78**	**0.06**
AGPathFinder with *ψ*(*Sc*,*Td*) = *ψ*(*false*,*true*)	0.64	0.65	0.63	0.64	0.15	0.71	0.72	0.70	0.71	0.07

The best performer for the relative item is marked in bold.

As can be seen from Tables 2 and 3, when we focus on the top path computed by each method, AGPathFinder showed improved performance compared to four other methods in most cases, the only exception is that the *Ac*, *Sn* and *Fm* values of AGPathFinder in the case of *ψ*(*Sc*,*Td*) = *ψ*(*false*, *true*) are a bit lower than those of RouteSearch, and the *Er* value of AGPathFinder in the case of *ψ*(*Sc*,*Td*) = *ψ*(*false*, *true*) is a bit higher than that of RouteSearch.

Regarding the performance of the best of top ten paths, in Table 2, we can see that AGPathFinder obtained higher values of *Ac*, *PPV*, *Sn* and *Fm* and lower values of *Er* than Tinker, LPAT and ReTrace whereas the performance of AGPathFinder is comparable with the performance of RouteSearch except for the sensitivity *Sn*. As can be seen from Table 3, in the cases of *ψ*(*Sc*,*Td*) = *ψ*(*true*, *true*) and *ψ*(*Sc*,*Td*) = *ψ*(*true*, *false*), AGPathFinder performs better than Tinker, RouteSearch and ReTrace with the highest values of *Ac_R*, *PPV_R*, *Sn_R*, *Fm_R* and the lowest values of *Er_R* while in the case of *ψ*(*Sc*,*Td*) = *ψ*(*true*, *false*), the performance of AGPathFinder is comparable with the performance of LPAT. In the case of *ψ*(*Sc*,*Td*) = *ψ*(*false*, *true*), the *Ac_R*, *PPV_R*, *Sn_R*, and *Fm_R* values of AGPathFinder is a bit weaker than those of RouteSearch Retrace and LPAT, and the *Ac*, *PPV*, *Sn*, *Fm* and *Er* values of AGPathFinder is a bit weaker than those of RouteSearch.

The results from Tables 2 and 3 demonstrate that inferring metabolic pathways by tracking atomic group and using combined information of reaction thermodynamics and compound similarity improves the quality of computed pathways. The ability of AGPathFinder in recovering known pathways is better than the other four methods.

Table 4 shows the values of *S-Paths* and *A-length* of the pathways computed by each method where *S-Paths* is the number of the computed pathways and *A-length* is the average length of the computed pathways.

**Table 4:** *S-Paths* and *A-length* of the pathways computed by each method.

Method	*S-Paths*	*A-length*
RouteSearch	412	5.23
Tinker	164	4.39
LPAT	321	5.78
ReTrace	381	4.25
AGPathFinder with *ψ*(*Sc*,*Td*) = *ψ*(*true*,*true*)	179	3.69
AGPathFinder with *ψ*(*Sc*,*Td*) = *ψ*(*true*,*false*)	170	3.67
AGPathFinder with *ψ*(*Sc*,*Td*) = *ψ*(*false*,*true*)	141	3.19

It can be seen from Table 4 that the average length of the pathways found by AGPathFinder is much shorter than the other four methods. Moreover, the pathways found by our method are more similar to the known metabolic pathways (Tables 2 and 3), that is, the pathways found by our method contain more reactions that are the same as in the known pathways. The reason for the shorter lengths of the pathways found by AGPathFinder is because the distances between reactions within the same metabolic pathway are significantly shorter than those between pairs of reactions selected at random [37] and more reactions in the pathways found by our method are involved in the same known pathways.

Note that, for the computed pathways in Table 4, no hub metabolites listed in [19] are involved in the computed pathways of AGPathFinder, Tinker, LPAT and ReTrace. However, some hub metabolites listed in [19] are involved in 272 out of 412 computed pathways of RouteSearch.

The above results demonstrate that compared with RouteSearch, Tinker, LPAT and ReTrace, AGPathFinder can find shorter pathways with better biochemical performance in general.

### The impact of parameter setting on the performance of AGPathFinder

In the following, we investigate the impact of the size of atomic group, the compound similarity and the reaction thermodynamics on the performance of AGPathFinder. The performance of AGPathFinder under different parameter settings is shown in Tables 5 and 6.

**Table 5:** Compound Inclusion Performance of AGPathFinder under different parameter settings.

Atomic group tracking	Compound similarity and reaction thermodynamics	Top Path				Best of top ten paths			
		*Ac*	*PPV*	*Sn*	*Fm*	*Ac*	*PPV*	*Sn*	*Fm*
Maximal size of atomic group		0.91	0.94	0.88	0.91	**0.95**	**0.97**	**0.94**	**0.95**
Minimal size of atomic group	*ψ*(*Sc*,*Td*) = *ψ*(*true*,*true*)	0.86	0.91	0.82	0.86	0.93	0.96	0.91	0.93
No atomic group		0.82	0.90	0.74	0.81	0.91	**0.97**	0.84	0.90
Maximal size of atomic group		**0.92**	**0.95**	**0.90**	**0.92**	0.94	0.96	0.92	0.94
Minimal size of atomic group	*ψ*(*Sc*,*Td*) = *ψ*(*true*,*false*)	0.80	0.90	0.83	0.86	0.93	0.95	0.91	0.93
No atomic group		0.80	0.89	0.72	0.80	0.90	0.96	0.84	0.90
Maximal size of atomic group		0.88	0.92	0.84	0.88	0.91	0.95	0.88	0.91
Minimal size of atomic group	*ψ*(*Sc*,*Td*) = *ψ*(*false*,*true*)	0.82	0.88	0.76	0.82	0.91	0.94	0.87	0.90
No atomic group		0.79	0.88	0.71	0.79	0.90	0.94	0.86	0.90

The best performer for the relative item is marked in bold.

**Table 6:** Reaction Inclusion Performance of AGPathFinder under different parameter settings.

Atomic group tracking	Compound similarity and reaction thermodynamics	Top Path				Best of top ten paths			
		*Ac_R*	*PPV_R*	*Sn_R*	*Fm_R*	*Ac_R*	*PPV_R*	*Sn_R*	*Fm_R*
Maximal size of atomic group		**0.70**	**0.71**	**0.69**	**0.70**	**0.78**	**0.78**	**0.77**	**0.78**
Minimal size of atomic group	*ψ*(*Sc*,*Td*) = *ψ*(*true*,*true*)	0.66	0.67	0.64	0.66	0.76	0.77	0.75	0.76
No atomic group		0.56	0.57	0.55	0.56	0.75	0.77	0.72	0.75
Maximal size of atomic group		**0.70**	0.70	**0.69**	**0.70**	**0.78**	**0.78**	**0.77**	**0.78**
Minimal size of atomic group	*ψ*(*Sc*,*Td*) = *ψ*(*true*,*false*)	0.66	0.67	0.65	0.66	**0.78**	**0.78**	**0.77**	**0.78**
No atomic group		0.54	0.55	0.52	0.54	0.74	0.77	0.71	0.74
Maximal size of atomic group		0.64	0.65	0.63	0.64	0.73	0.73	0.72	0.72
Minimal size of atomic group	*ψ*(*Sc*,*Td*) = *ψ*(*false*,*true*)	0.54	0.55	0.53	0.54	0.72	0.73	0.70	0.72
No atomic group		0.49	0.50	0.47	0.49	0.71	0.72	0.70	0.71

The best performer for the relative item is marked in bold.

Tables 5 and 6 show that, for each setting of *ψ*(*Sc*,*Td*), the computed pathways that conserve the maximal size of atomic group have the highest values of *Ac*, *Sn*, *PPV*, *Fm*, *Ac_R*, *PPV_R*, *Sn_R*, and *Fm_R*. This confirms that the transfer of atomic groups from the start compound to the target compound is an important feature for metabolic pathways, and the size of an atomic group has direct impact on the biochemical performance of the pathways found by our method.

It can also be observed from Tables 5 and 6 that the parameter setting of *ψ*(*Sc*,*Td*) directly influences the performance of AGPathFinder. For example, when we search pathways by tracking “Maximal size of atomic group”, for the top path, the setting *ψ*(*Sc*,*Td*) = *ψ*(*true*,*false*) gives the highest values of *Ac*, *Sn*, *PPV* and *Fm*, and the setting *ψ*(*Sc*,*Td*) = *ψ*(*true*,*true*) gives the highest values of *Ac_R*, *PPV_R*, *Sn_R*, and *Fm_R*. While the setting *ψ*(*Sc*,*Td*) = *ψ*(*true*,*true*) produces the highest values of *Ac*, *Sn*, *PPV*, *Fm*, *Ac_R*, *PPV_R*, *Sn_R*, and *Fm_R* in the best of top ten paths.

Moreover, the use of combined information of compound similarity and reaction thermodynamics ensures that our method is still capable in finding meaningful pathways even without the option of tracking atomic groups. For example, without atomic group tracking and from a total of 42 known pathways in the test, AGPathFinder successfully recovered 28, 27 and 28 known pathways that are returned as the best of top ten paths in the cases of *ψ*(*Sc*,*Td*) = *ψ*(*false*,*true*), *ψ*(*Sc*,*Td*) = *ψ*(*true*,*false*) and *ψ*(*Sc*,*Td*) = *ψ*(*true*,*true*) respectively, and recovered 18, 21 and 22 known pathways that are returned as the top path in the cases of *ψ*(*Sc*,*Td*) = *ψ*(*false*,*true*), *ψ*(*Sc*,*Td*) = *ψ*(*true*,*false*) and *ψ*(*Sc*,*Td*) = *ψ*(*true*,*true*) respectively.

### Study cases

The above analysis clearly demonstrates that our method AGPathFinder improves the biochemical relevance of the computed pathways. In order to investigate the factors that may influence the biochemical relevance of the found pathways, we perform three representative test cases to analyze the results obtained by AGPathFinder, RouteSearch, Tinker, LPAT and ReTrace. The aim of this section is not to demonstrate the average or overall performance of pathfinding (these were already discussed in section “Comparing computed pathways to known pathways”), but to gain insight into the characteristics of the methods through analysis.

#### L-serine biosynthesis

The biosynthesis of L-serine starts with 3-phospho-D-glycerate to L-serine. 3-phospho-D-glycerate contains 11 atoms, 3 of which are carbons, and L-serine contains 7 atoms, 3 of which are carbons. An atomic group containing 5 atoms (C, C, C, O, O) is transferred from 3-phospho-D-glycerate to L-serine in the biosynthesis of L-serine.

**Fig 4:**
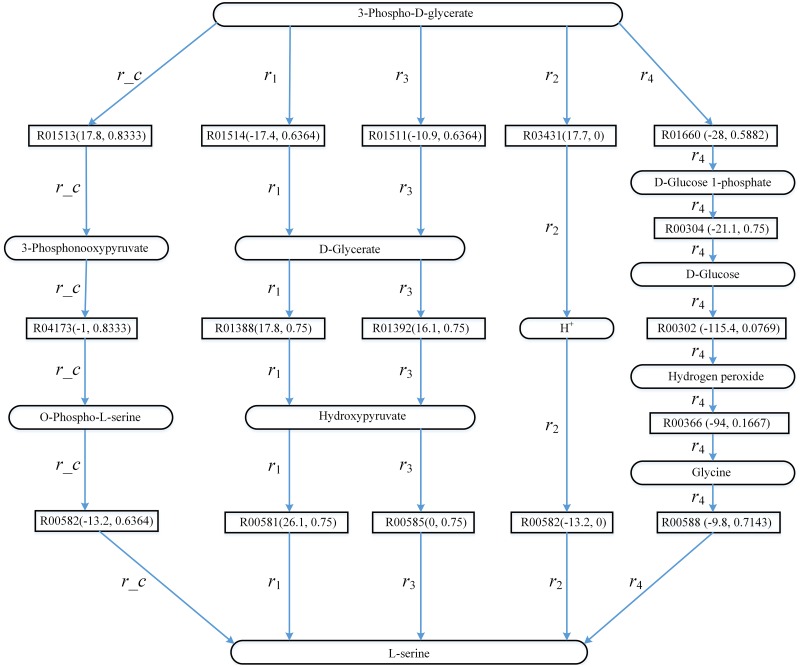
Computed pathways for L-serine biosynthesis: *r*_1_, *r*_2_, *r*_3_ and *r*_4_. Rectangles represent reaction edges, and the data in parentheses denote the value $\Delta {G^{\prime }}_{r}$ of reaction and the compound similarity respectively.

Fig 4 shows the known pathway *r_c* (the biosynthesis of L-serine in KEGG) and the pathways found by AGPathFinder, RouteSearch, Tinker, LPAT and ReTrace. The top ranking pathway returned by LPAT is *r*_1_ in KEGG. For all three settings *ψ*(*Sc*,*Td*) = *ψ*(*true*,*true*), *ψ*(*Sc*,*Td*) = *ψ*(*true*,*false*) and *ψ*(*Sc*,*Td*) = *ψ*(*false*,*true*), all the top ranking pathways found by AGPathFinder are *r_c* in KEGG. The top ranking pathway returned by RouteSearch is *r*_2_ in the EcoCyc database [55]. The top ranking pathway returned by ReTrace is *r*_3_ in KEGG. The top ranking pathway returned by Tinker is *r*_4_ in the Rhea database [56].

**Fig 5:**
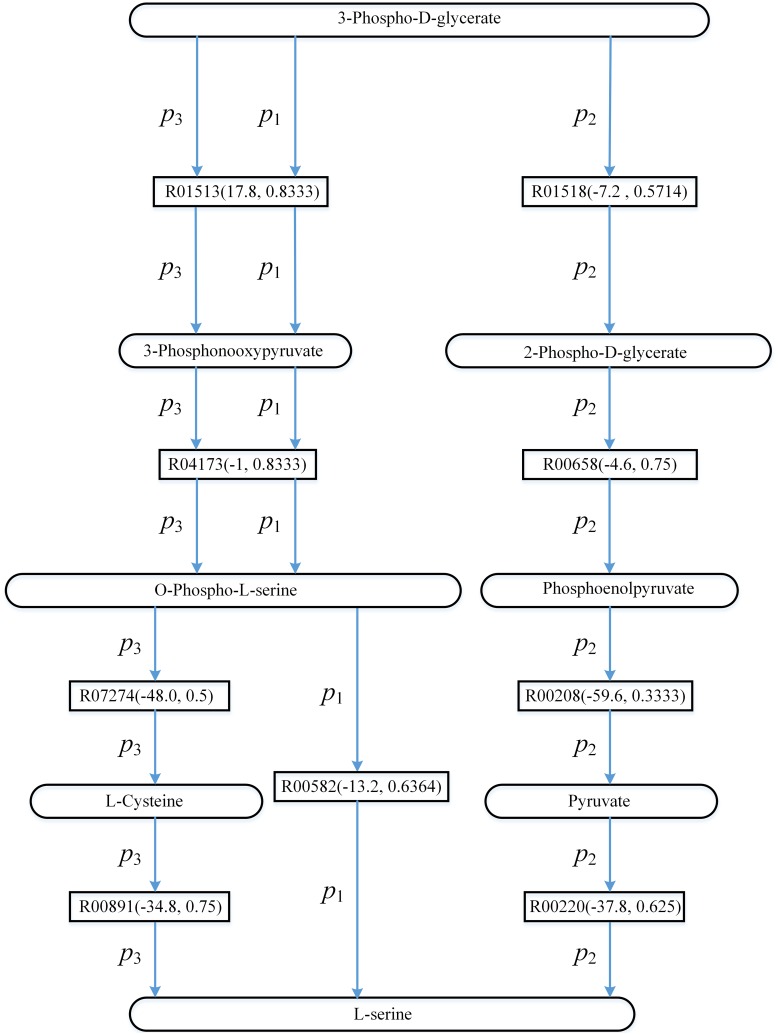
The top three pathways found by AGPathFinder in the search of L-serine biosynthesis in KEGG when *ψ*(*Sc*,*Td*) = *ψ*(*false*,*true*) (these three pathways are represented by *p*_1_, *p*_2_ and *p*_3_ respectively). Rectangles represent reaction edges, and the data in parentheses denote the value $\Delta {G^{\prime }}_{r}$ of reaction and the compound similarity respectively.

Recall that if *ψ*(*Sc*,*Td*) = *ψ*(*false*,*true*), the search of the pathways will be driven by Gibbs free energy of the reactions. To investigate the impacts of the utilization of energy on finding pathways from 3-phospho-D-glycerate to L-serine, Fig 5 shows the top three pathways found by AGPathFinder in the search of L-serine biosynthesis in KEGG when *ψ*(*Sc*,*Td*) = *ψ*(*false*,*true*). These three pathways are represented by *p*_1_, *p*_2_ and *p*_3_ respectively.

As we can observe from Fig 4, compared with pathways *r*_1_, *r*_2_, *r*_3_ and *r*_4_, the pathway *r_c* for the biosynthesis of L-serine consists of reactions with minimal $\Delta {G^{\prime }}_{r}$ and highly similar compounds. By tracking atomic group and using the combined information of compound similarity and reaction thermodynamics, our method found *r_c* in all cases with different settings of *ψ*(*Sc*,*Td*). AGPathFinder is thus useful for retrieving known metabolic pathways. In addition, *r*_1_, *r*_2_, *r*_3_ and *r*_4_ are obviously different from *r_c* except for the start and target compounds. *r*_2_ is the shortest, but hub metabolite H^+^ occurs in its pathway.

In Fig 5, the search of the pathways is driven by Gibbs free energy of the reactions and the pathways that involve the reactions with low energy are ranked ahead. For example, compared with pathway *p*_3_, the energies of the corresponding reactions in pathway *p*_2_ are lower and therefore *p*_2_ is ahead of *p*_3_. Note that our method is a shortest-path-based method, although the energies of the reactions in pathway *p*_1_ are not the lowest, *p*_1_ is the top ranking pathway since it is the shortest pathway. Moreover, the energies of the reactions in *p*_2_ are negative. This indicates that when we try to find the pathways releasing energy from 3-phospho-D-glycerate to L-serine, we can choose *p*_2_. On the other hand, the energies of the reactions in *p*_1_ and *p*_3_ can be negative or positive, and the user can find the pathways that either require or release energy such as *p*_1_ and *p*_3_.

#### Glycolysis

Glycolysis starts from beta-D-Fructose 6-phosphate to phosphoenolpyruvate in aMAZE [52]. Compound beta-D-Fructose 6-phosphate contains 16 atoms, 6 of which are carbons, and phosphoenolpyruvate contains 10 atoms, 3 of which are carbons. An atomic group containing 9 atoms (P, O, O, O, O, C, C, C, O) is transferred from beta-D-Fructose 6-phosphate to phosphoenolpyruvate in glycolysis.

**Fig 6:**
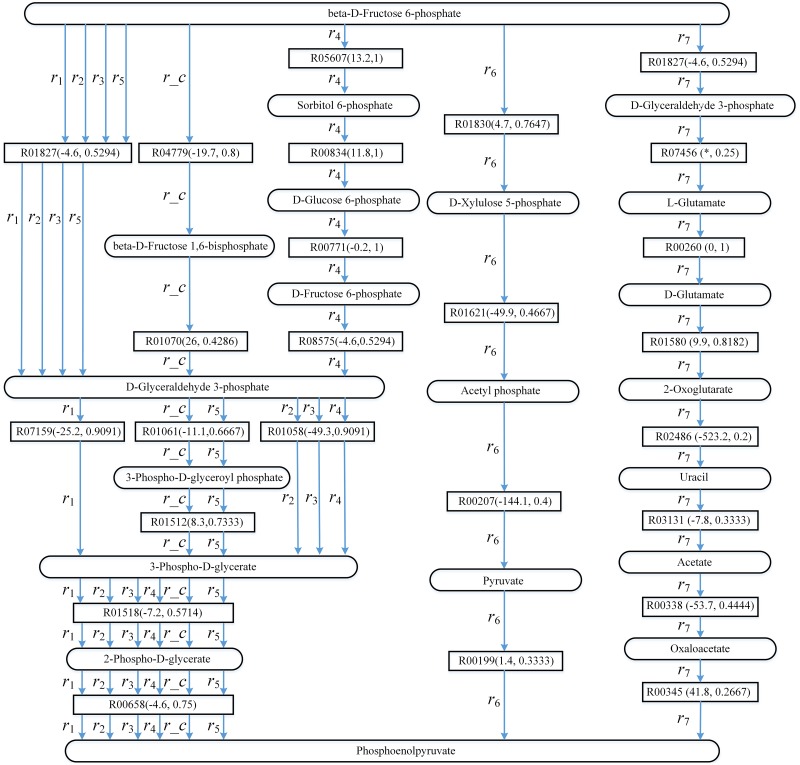
Computed pathways for glycolysis: *r*_1_, *r*_2_, *r*_3_, *r*_4_, *r*_5_, *r*_6_ and *r*_7_. Round rectangles represent compound nodes, rectangles represent reaction edges, and the data in parentheses denote the value $\Delta {G^{\prime }}_{r}$ of reaction and compound similarity respectively. “*” means that the Gibbs free energy of the corresponding reaction is not available.

Fig 6 shows the known pathway *r_c* (glycolysis in aMAZE) and the pathways found by AGPathFinder, RouteSearch, Tinker, LPAT and ReTrace. The top ranking pathway returned by LPAT is *r*_1_ in KEGG. The top ranking pathways returned by AGPathFinder are *r*_2_ and *r*_3_ in KEGG when *ψ*(*Sc*,*Td*) = *ψ*(*true*,*true*) and *ψ*(*Sc*,*Td*) = *ψ*(*false*,*true*) respectively. The top ranking pathway returned by AGPathFinder is *r*_4_ in KEGG when *ψ*(*Sc*,*Td*) = *ψ*(*true*,*false*). The top ranking pathway returned by RouteSearch is *r*_5_ in EcoCyc. The top ranking pathway returned by ReTrace is *r*_6_ in KEGG. The top ranking pathway returned by Tinker is *r*_7_ in Rhea.

**Fig 7:**
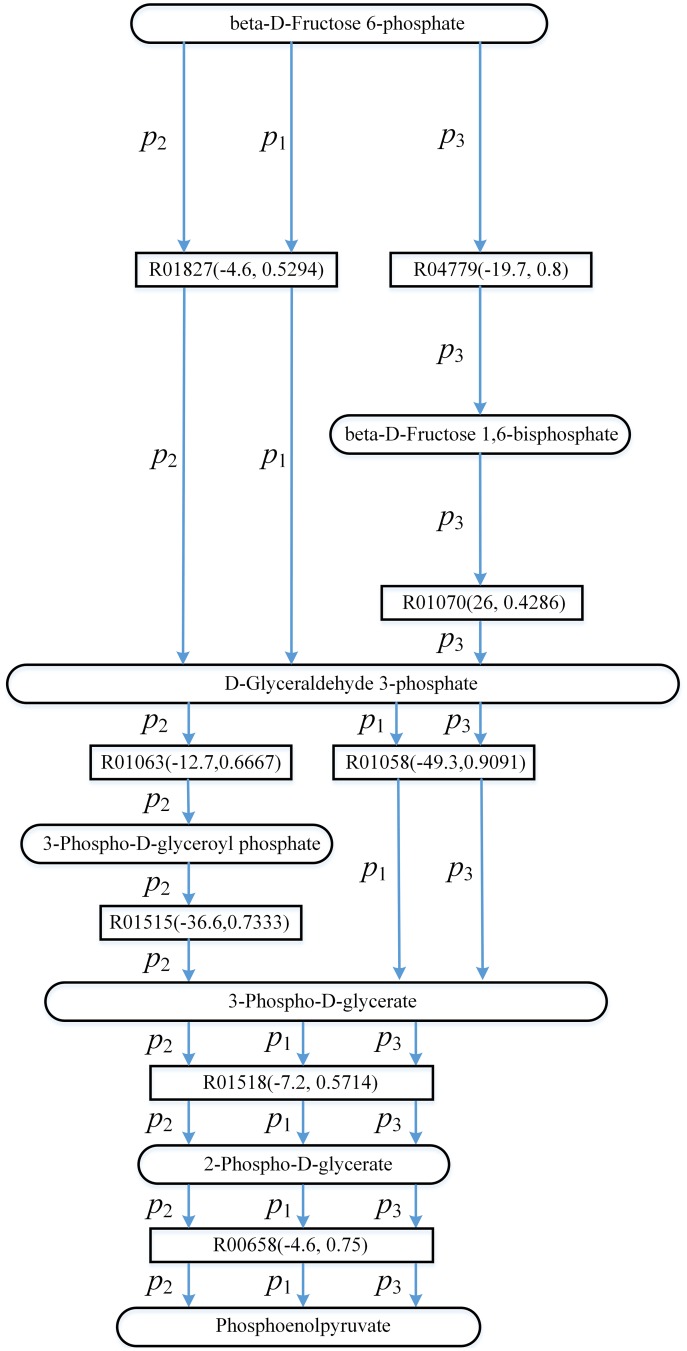
The top three pathways found by AGPathFinder in the search of glycolysis in KEGG when *ψ*(*Sc*,*Td*) = *ψ*(*false*,*true*) (these three pathways are represented by *p*_1_, *p*_2_ and *p*_3_ respectively). Round rectangles represent compound nodes, rectangles represent reaction edges, and the data in parentheses denote the value $\Delta {G^{\prime }}_{r}$ of reaction and compound similarity respectively.

To study the impacts of the utilization of energy on finding pathways from beta-D-Fructose 6-phosphate to phosphoenolpyruvate, Fig 7 shows the top three pathways found by AGPathFinder in the search of glycolysis in KEGG when *ψ*(*Sc*,*Td*) = *ψ*(*false*,*true*). These three pathways are represented by *p*_1_, *p*_2_ and *p*_3_ respectively.

As can be seen from Fig 6, the similarity between two compounds involved in each reaction in Fig 6 is high, most of these similarities are higher than 0.4. We can observe that *r*_5_ is similar to *r_c*, and their difference is that *r*_5_ does not bypass beta-D-Fructose 1,6-bisphosphate. Compared to *r_c*, the pathways *r*_1_, *r*_2_ and *r*_3_ do not go through beta-D-Fructose 1,6-bisphosphate and 3-Phospho-D-glyceroyl phosphate. In addition, *r*_2_ and *r*_3_ consist of an alternative pathway connecting beta-D-Fructose 6-phosphate with D-Glyceraldehyde 3-phosphate via reaction R01827. This alternative pathway is shorter than the pathway between beta-D-Fructose 6-phosphate and D-Glyceraldehyde 3-phosphate in *r_c*.

Furthermore, in *r*_1_, *r*_2_, *r*_3_ and *r*_4_, there is a shortcut linking D-Glyceraldehyde 3-phosphate and 3-Phospho-D-glycerate via R07159 and R01058 respectively, these shortcuts are annotated in the corresponding KEGG map00010 (Glycolysis/Gluconeogenesis). The difference between *r*_4_ and *r_c* is large except for the pathway from 3-Phospho-D-glycerate to Phosphoenolpyruvate. *r*_4_ goes through the pathway from beta-D-Fructose 6-phosphate to D-Glyceraldehyde 3-phosphate as a result of the high similarities between the compounds contained in this part of *r*_4_, for example, both similarity between beta-D-Fructose 6-phosphate and Sorbitol 6-phosphate and similarity between D-Glucose 6-phosphate and D-Fructose 6-phosphate are 1. For the same reason, *r*_4_ bypasses the pathway from D-Glyceraldehyde 3-phosphate to 3-Phospho-D-glycerate. This indicates that one can use compound similarity to filter pathway assignments for feasibility.

In addition, none of the reactions and compounds in *r*_6_ and *r*_7_ is common with those of *r_c*, except for the start and target compounds. We can also see that, *r*_2_ and *r*_3_ are very similar to *r*_1_, and they only differ in one reaction. These results show that AGPathFinder and LPAT are capable in finding similar alternative pathways of glycolysis.

Seen from Fig 7, pathway *p*_1_ is the shortest pathway and therefore it is the top ranking pathway. Although the length of pathways *p*_2_ and *p*_3_ is the same, *p*_2_ is ahead of *p*_3_ since the sum of the energies of the reactions in *p*_2_ is lower. In *p*_1_ and *p*_2_, the energies of the reactions are negative, which demonstrates that we can search for the pathways releasing energy such as *p*_1_ and *p*_2_ from beta-D-Fructose 6-phosphate to phosphoenolpyruvate. In *p*_3_, the energy of reaction R01070 is positive whereas the energies of other reactions are negative, thus we can find the pathway that either require or release energy like *p*_3_.

#### L-Methionine biosynthesis

The biosynthesis of L-Methionine starts with L-Aspartate to L-Methionine. L-Aspartate contains 9 atoms, 4 of which are carbons, and L-Methionine contains 9 atoms, 5 of which are carbons. Two variants of this pathway are characterized in the yeast *S*. *cerevisiae* and the bacteria *E*. *coli*, respectively. An atomic group with 7 atoms (C, C, C, N, O, O, C) is transferred from L-Aspartate to L-Methionine in the biosynthesis of L-Methionine.

**Fig 8:**
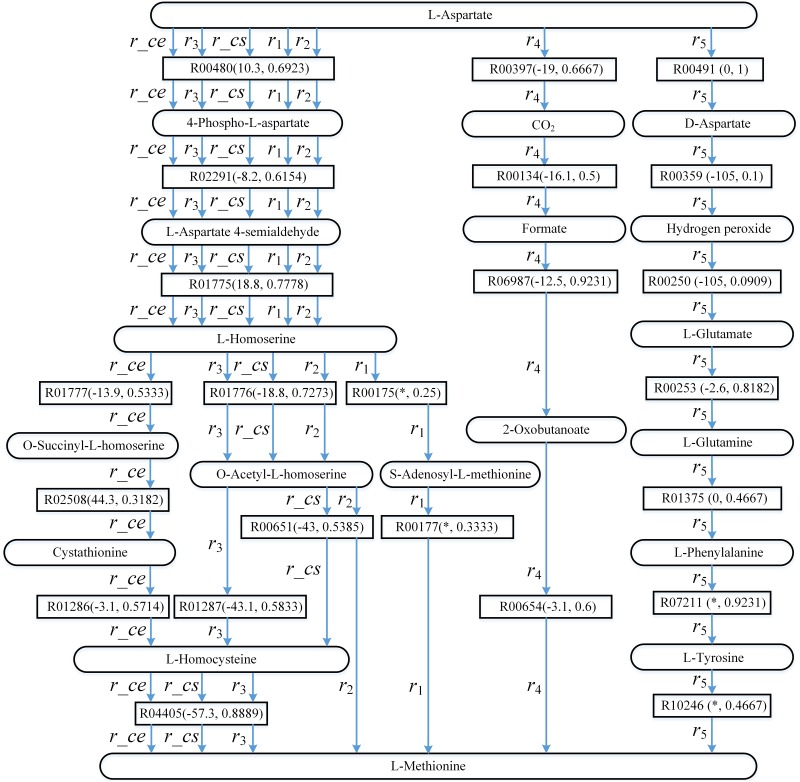
Computed pathways for L-Methionine biosynthesis: *r*_1_, *r*_2_, *r*_3_, *r*_4_ and *r*_5_. Round rectangles represent compound nodes, rectangles represent reaction edges, and the data in parentheses denote the value $\Delta {G^{\prime }}_{r}$ of reaction and compound similarity respectively. “*” means that the Gibbs free energy of the corresponding reaction is not available.

Fig 8 shows the known pathways *r_ce* (the biosynthesis of L-Methionine for bacteria *E*. *coli* in aMAZE) and *r_cs* (the L-Methionine biosynthesis pathway for the yeast *S*. *cerevisiae* in aMAZE), and the pathways found by AGPathFinder, RouteSearch, Tinker, LPAT and ReTrace. The top ranking pathway returned by RouteSearch is *r_ce* in EcoCyc for *E*. *coli* K-12 substr. MG1655. The top ranking pathway returned by LPAT is *r*_1_ in KEGG. For all three settings of *ψ*(*Sc*,*Td*), the top ranking pathway returned by AGPathFinder is *r*_2_ in KEGG. Note that *r*_2_ is the only pathway found by AGPathFinder in the case of *ψ*(*Sc*,*Td*) = *ψ*(*false*,*true*). The top ranking pathway returned by RouteSearch is *r*_2_ in EcoCyc for *S*. *cerevisiae*. The second-ranked pathway returned by AGPathFinder is *r*_3_ in KEGG with the setting *ψ*(*Sc*,*Td*) = *ψ*(*true*,*true*). The top ranking pathway returned by ReTrace is *r*_4_ in KEGG. The top ranking pathway returned by Tinker is *r*_5_ in Rhea.

From Fig 8 it can be observed that, *r*_2_ corresponds closely to *r_cs*, and their difference is that *r*_2_ does not go through L-Homocysteine and reaction R04405. Because *r*_2_ via reaction R00651 is shorter, AGPathFinder chooses reaction R00651 in the process of inferring *r*_2_. In *r*_2_, the energies of the reactions can be negative or positive, and we can find the pathway that either require or release energy like *r*_2_ in the case of *ψ*(*Sc*,*Td*) = *ψ*(*false*,*true*).

Alternative pathway *r*_3_ is very similar to *r_cs* except for one reaction. The value of $\Delta {G^{\prime }}_{r}$ of R01287 in *r*_3_ is lower than the value of $\Delta {G^{\prime }}_{r}$ of R00651. Therefore, AGPathFinder chooses reaction R01287 in the process of inferring *r*_3_. Furthermore, *r*_1_ is similar to *r_cs*, but their difference is far greater than the difference between *r*_3_ and *r_cs*. In addition, *r*_4_ and *r*_5_ are completely different from *r_ce* and *r_cs* except for the start and target compounds. These alternative pathways demonstrate how AGPathFinder and other four methods can be used to expand the metabolism of L-Methionine synthesis. Through efficient search in the extensive spaces in designing synthetic metabolic pathways, the computational pathfinding methods can find new pathways producing the same target compound through different mechanisms than those already known. These pathways need to be further tested for biological and biochemical consistency before implementation. However, the results show promising alternatives to generate valuable products.

## Discussion and Conclusion

This article presents a pathfinding method AGPathFinder for finding metabolic pathways. The main feature of AGPathFinder is its integration of atomic group tracking and combined information of reaction thermodynamics and compound similarity into the search of metabolic pathways. This feature distinguishes AGPathFinder from existing atom tracking pathfinding methods, which are restricted to track the user-defined atoms in the search for alternative pathways.

In section “Results”, in most cases, we have shown that the average compound inclusion accuracy and reaction inclusion accuracy for the top resulting pathways of our method are around 0.90 and 0.70, respectively, which are better than those of RouteSearch, Tinker, LPAT and ReTrace. Atomic group tracking, when combined with weighted metabolite graph, improves the quality of the found pathways. With the introduction of atomic group tracking, our method does not require the user to define the atoms to be tracked neither to exclude hub metabolites in advance. On the other hand, the use of combined information of reaction thermodynamics and compound similarity ensures that our method is still capable in finding meaningful pathways even without the option of tracking atomic groups. The results have demonstrated that AGPathFinder successfully recovers the known pathways and finds the thermodynamically feasible pathways that avoid spurious connections. Moreover, AGPathFinder allows the user to define biochemical parameters to search for specific pathways of interest, and it returns pathways with the information of reaction thermodynamics and compound similarity.

AGPathFinder infers pathways across all of the data in KEGG, but for some applications, researchers may be only interested in the metabolic network of a single organism or several related organisms. A possible solution for this issue is to use alternative weighting schemes. For example, we try to find organism-specific pathways by weighting the reactions depending on the possibility that the organism of interest performs the corresponding reactions. This may help to find feasible candidates for *in vivo* experimentation.

In the current work, we have not considered the substrate availability or toxicity, and did not take the different reaction conditions such as pH and temperature into account when estimating thermodynamics. In future work, we intend to include these factors into AGPathFinder. Another interesting extension is to combine constraint programming methods with atomic group tracking in searching metabolic pathway.

## Availability of the Software

AGPathFinder is fully implemented in Java. The data and the program can be downloaded from http://210.36.16.170:8080/AGPathFinder/data.zip.

AGPathFinder is also available as a web service at http://210.36.16.170:8080/AGPathFinderWeb/login.jsp.

## Supporting Information

S1 Text The test set of metabolic pathways. (PDF)

S1 Table The hub metabolites listed in Tinker. (DOC)
