# Supplementary material for: A Method for Finding Metabolic Pathways Using Atomic Group Tracking
Source: PLoS One. 2017 Jan 9;12(1):e0168725. doi: 10.1371/journal.pone.0168725 (PMC5221824; doi:10.1371/journal.pone.0168725)
Supplement: S1 Text — (PDF) [file pone.0168725.s001.pdf]

S1 Text:

The test set of metabolic pathways

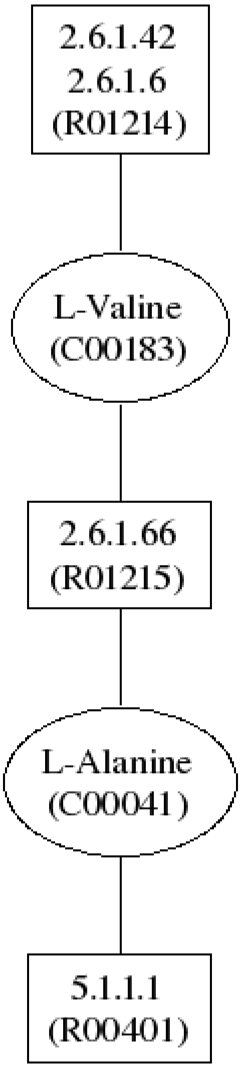

S1 Fig. Alanine Biosynthesis-Ecoli-C00183 C00041

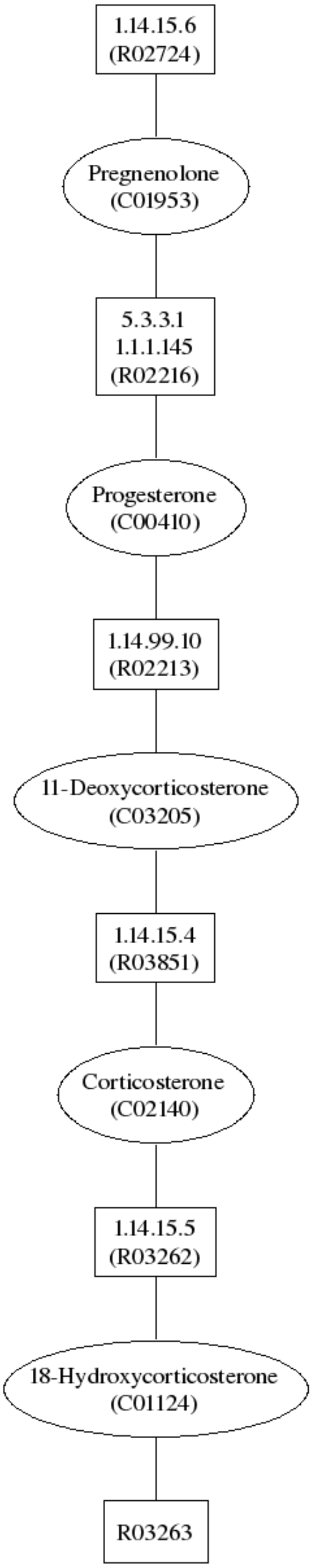

S2 Fig. Aldosterone Biosynthesis-Human-C01953 C01124

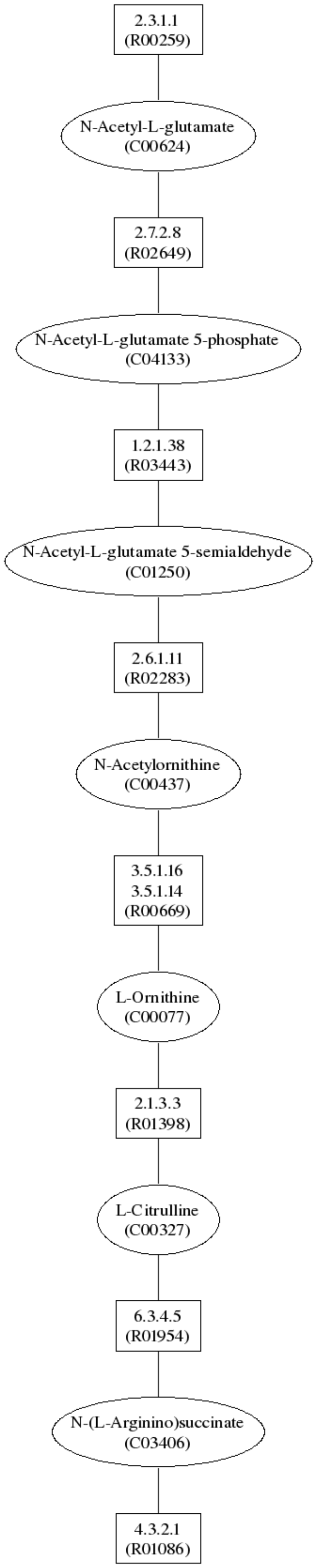

S3 Fig. Arginine biosynthesis-Ecoli-C00624 C03406

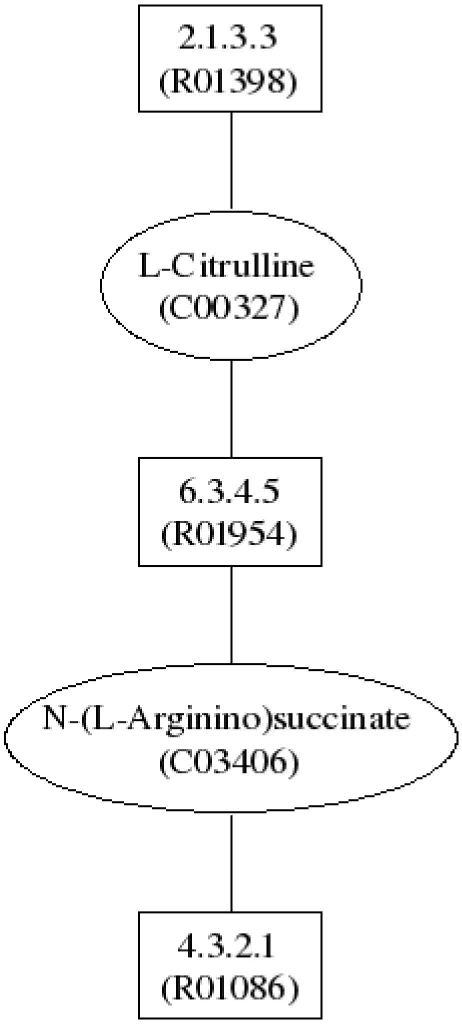

S4 Fig. Arginine Biosynthesis I-Human-C00327 C03406

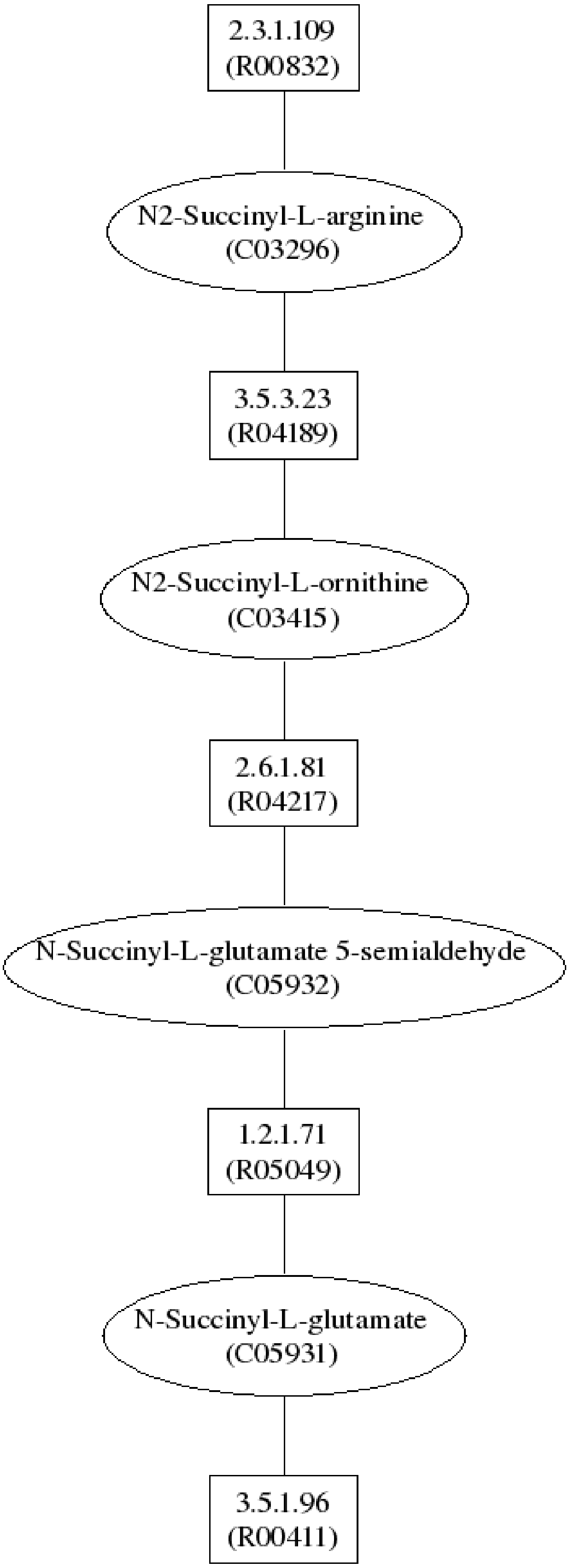

S5 Fig. Arginine Catabolism-Ecoli-C03296 C05931

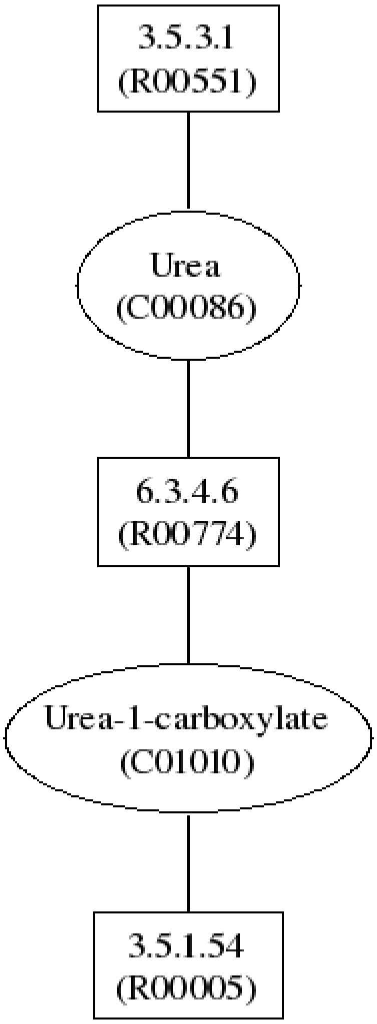

S6 Fig. Arginine Degradation-Yeast-C00086 C01010

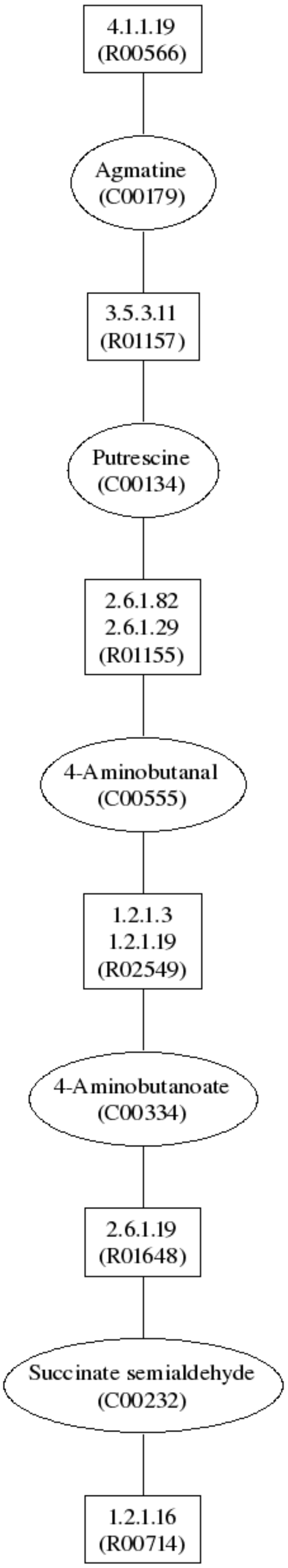

S7 Fig. Arginine Utilization-Ecoli-C00179 C00232

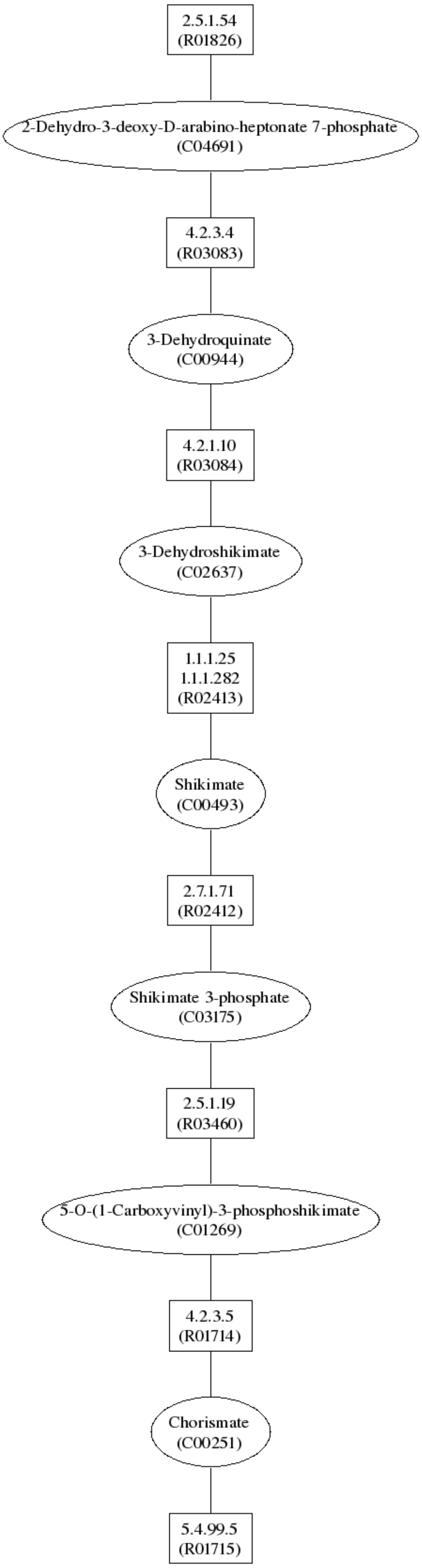

S8 Fig. Aromatic Amino Acid Path-Yeast-C04691 C00251

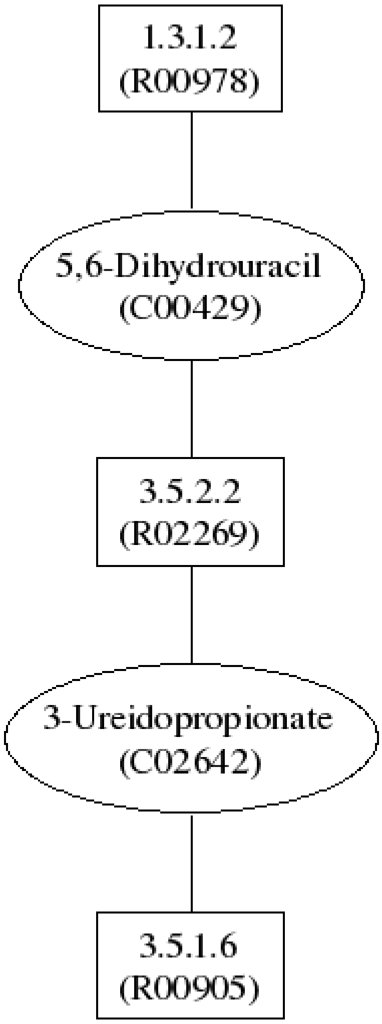

S9 Fig. BetaAlanine-Human-C00429 C02642

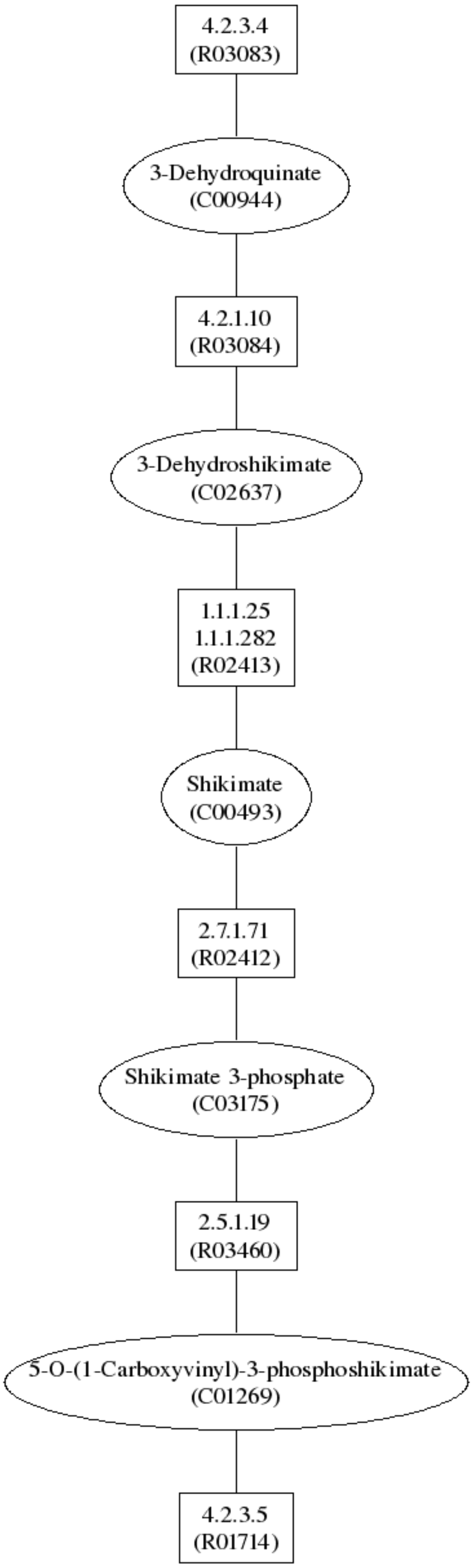

S10 Fig. Chorismate Biosynthesis-Ecoli-C00944 C01269

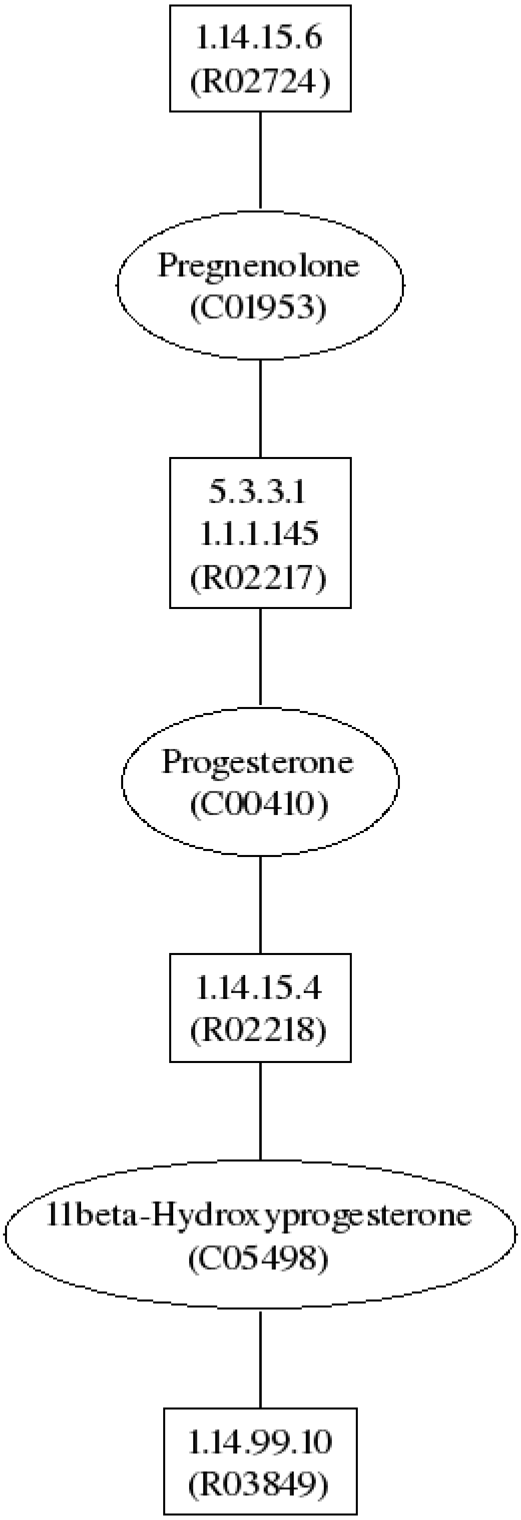

S11 Fig. Corticosterone Biosynthesis-Human-C01953 C05498

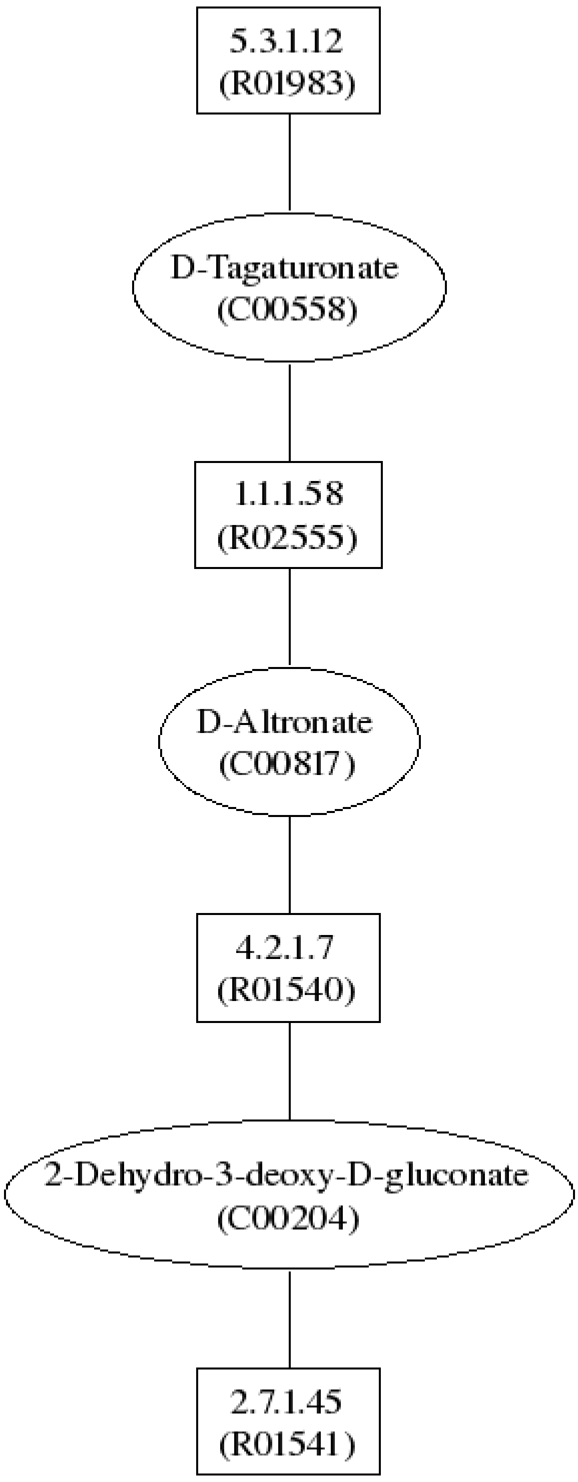

S12 Fig. D-galacturonate Catabolism-Ecoli-C00558 C00204

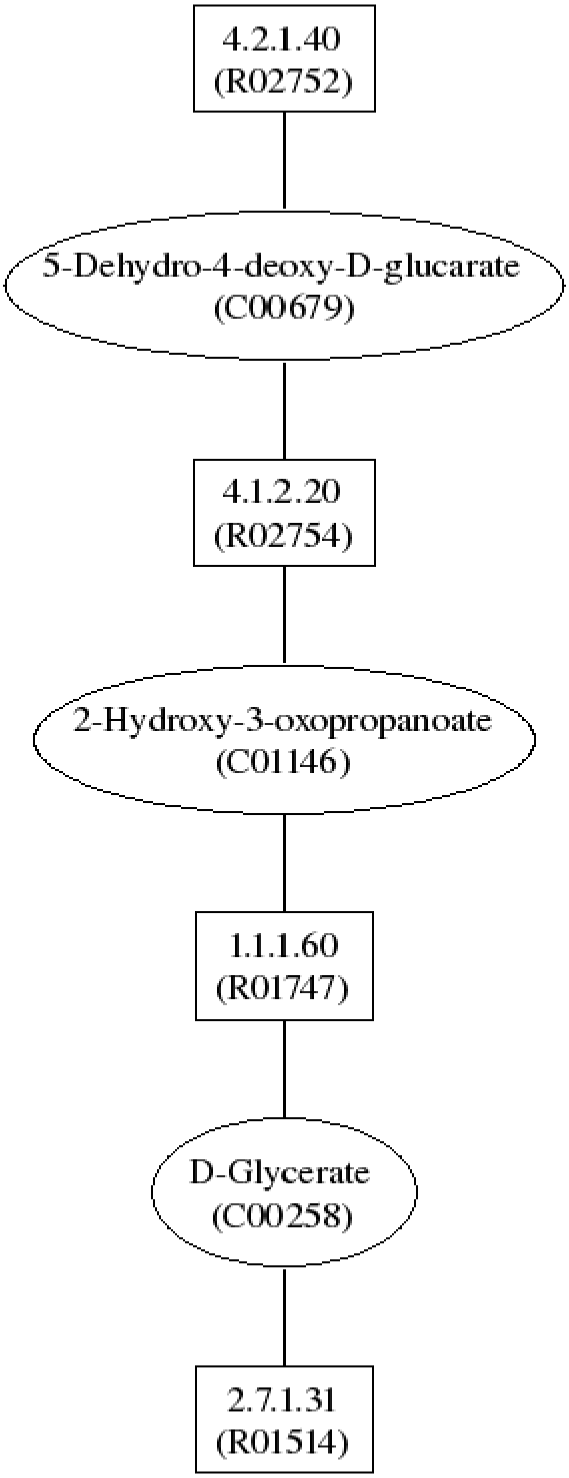

S13 Fig. D-glucarateand D-galactarate Catabolism

Super-Pathway-Ecoli-C00679 C00258

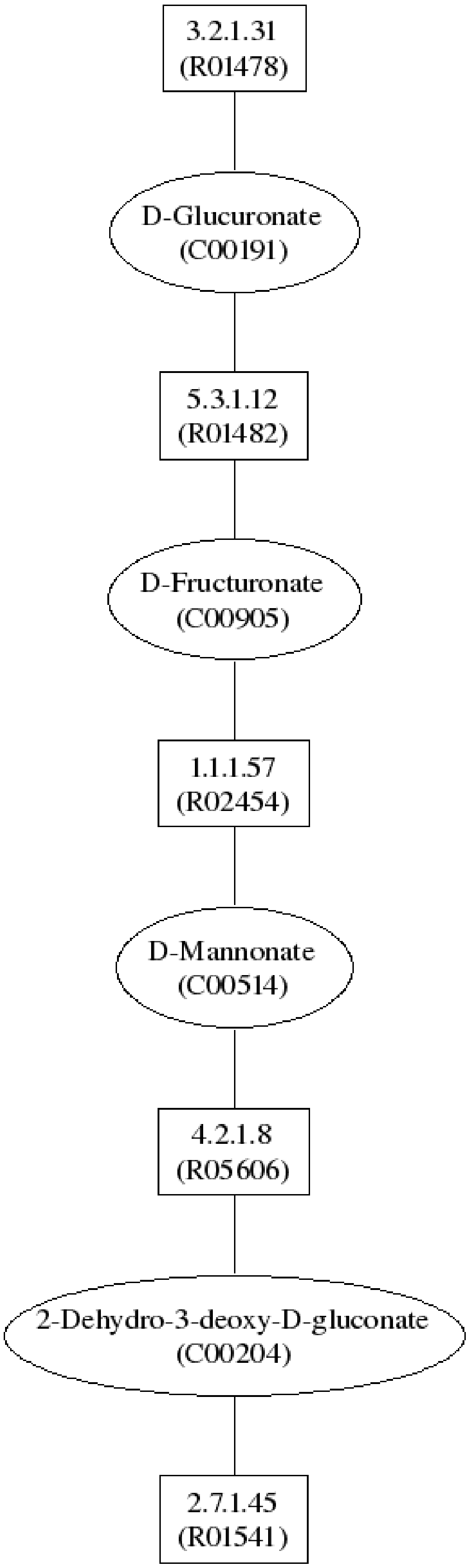

S14 Fig. D-Glucuronate Catabolism-Ecoli-C00191 C00204

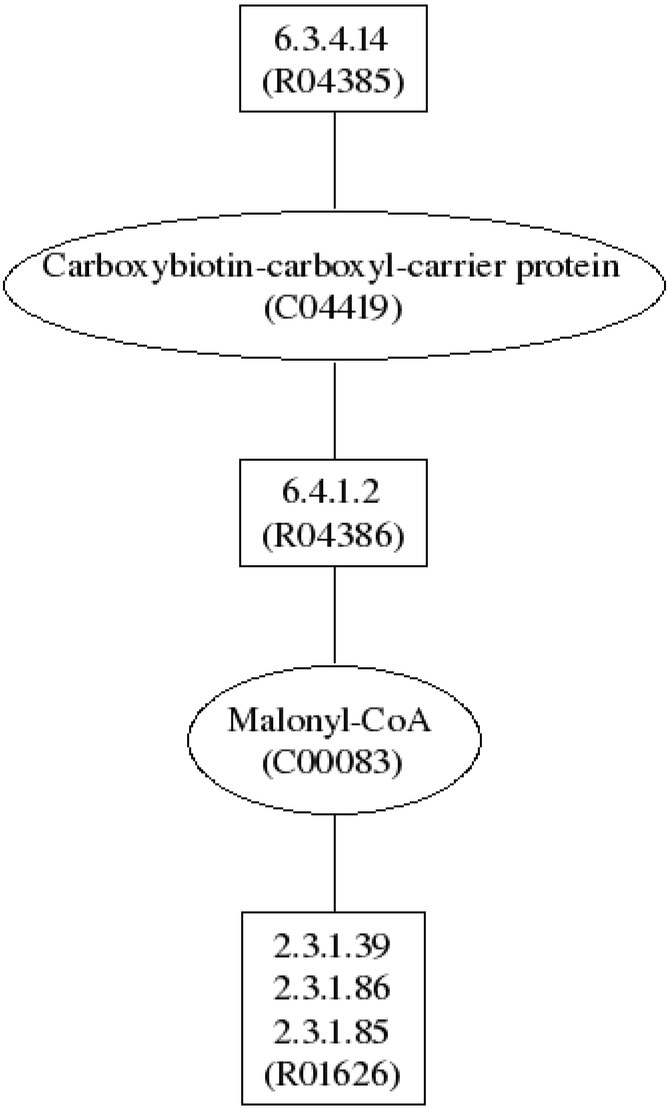

S15 Fig. Fatty acid Biosynthesis-Path I-Human-C04419 C00083

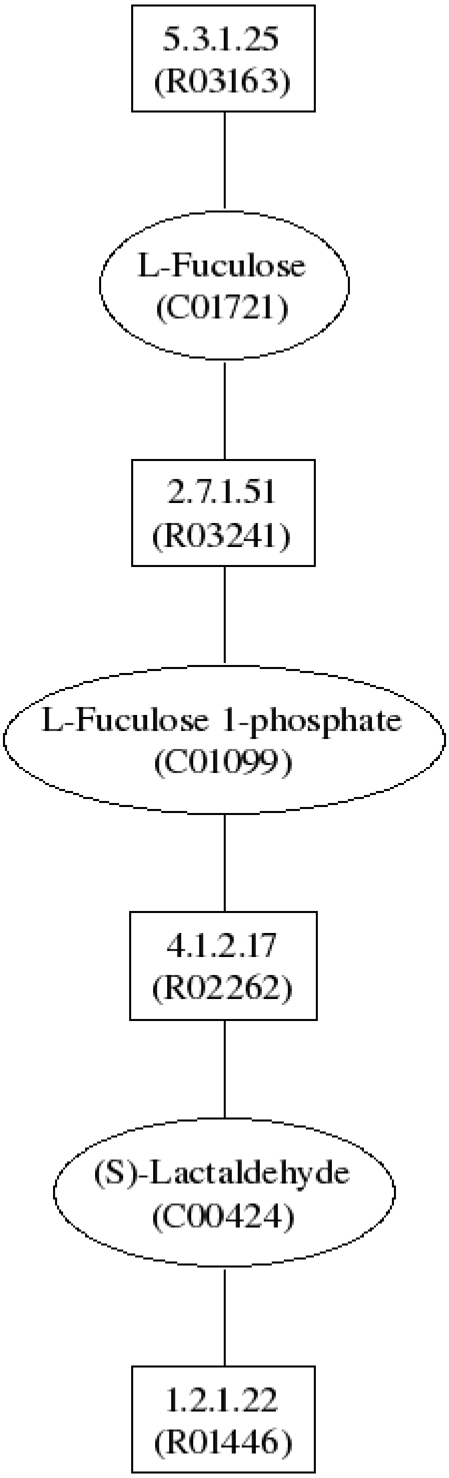

S16 Fig. Fucose Catabolism-Ecoli-C01721 C00424

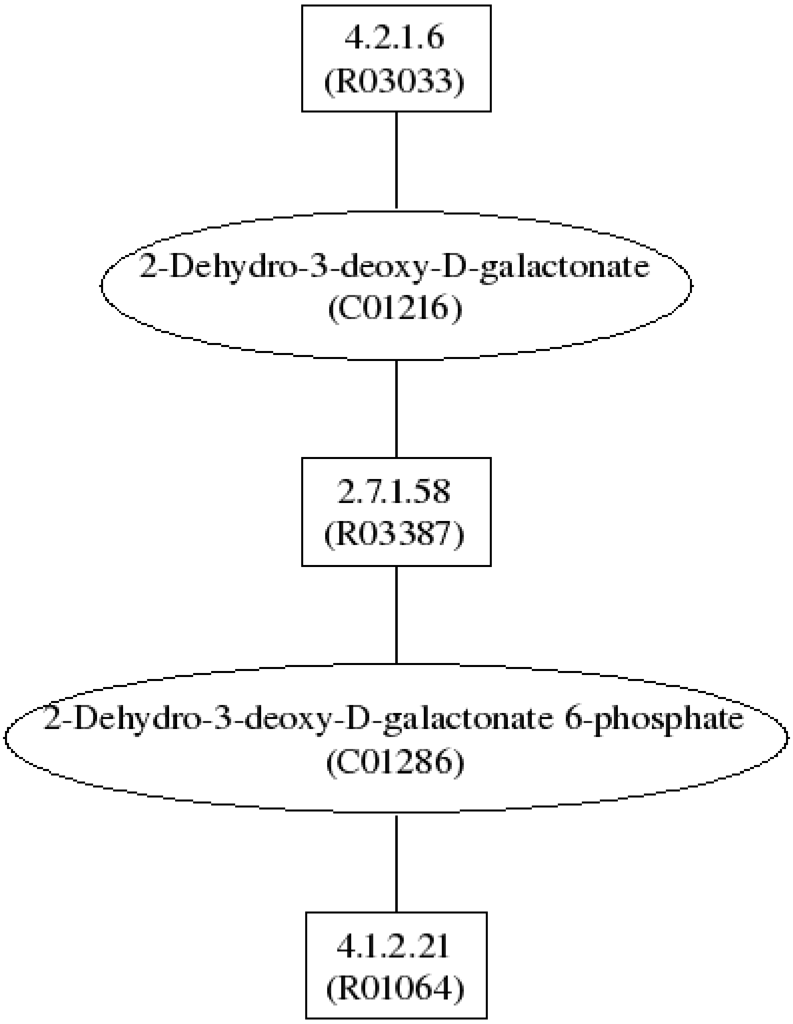

S17 Fig. Galactonate Catabolism-Ecoli-C01216 C01286

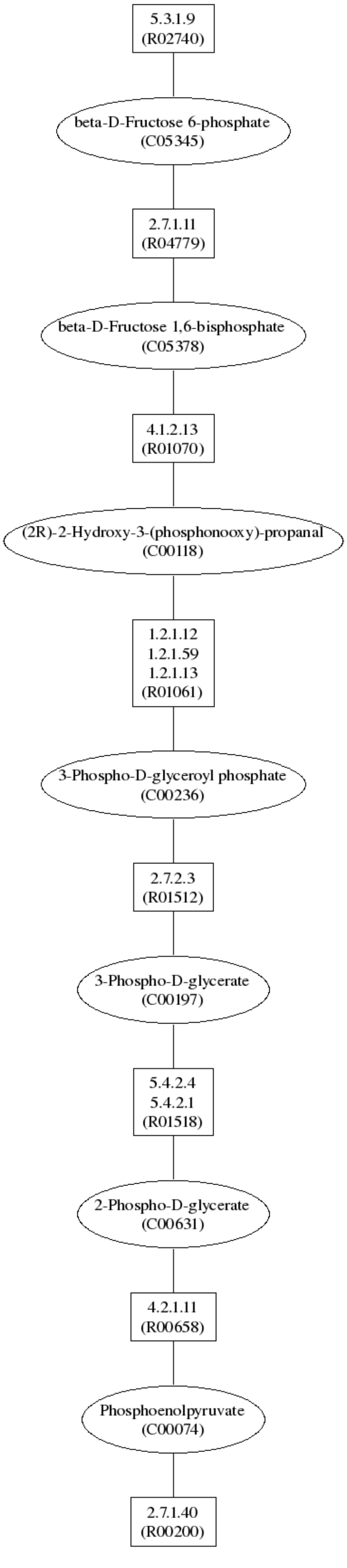

S18 Fig. Glycolysis-Ecoli-C05345 C00074

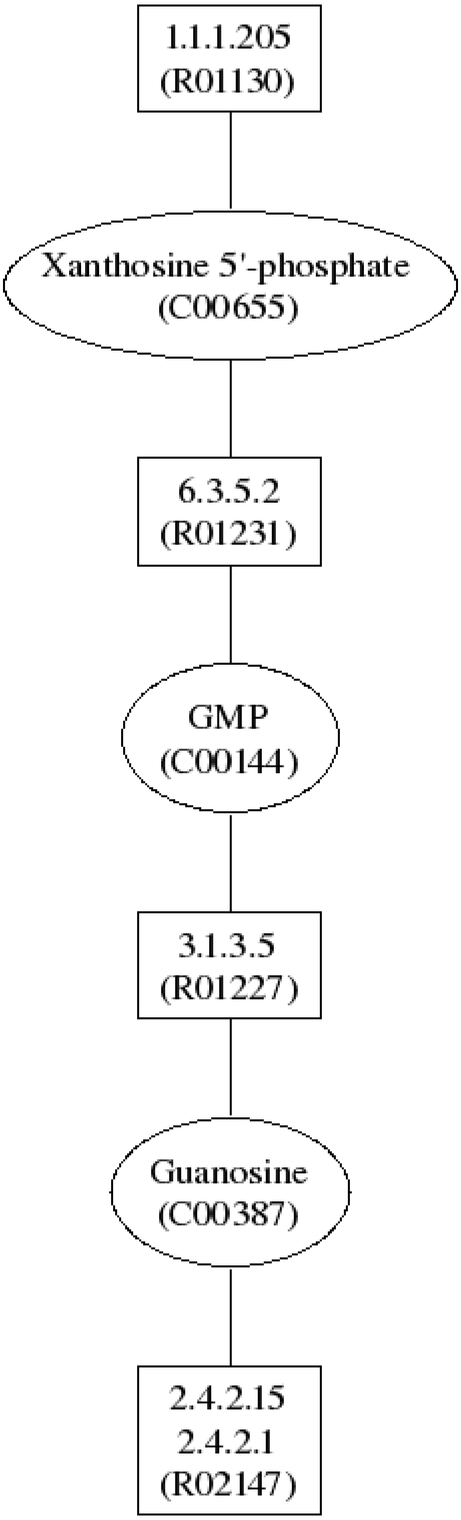

S19 Fig. Guanine Biosynthesis-Human-C00655 C00387

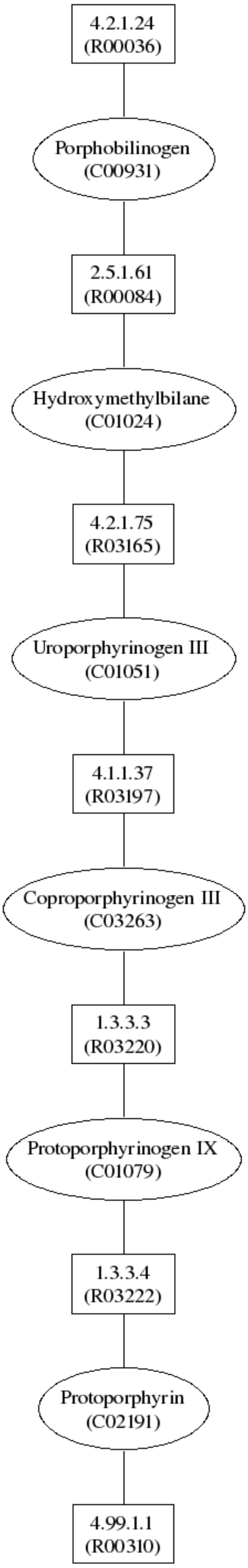

S20 Fig. Heme Biosynthesis-Human-C00931 C02191

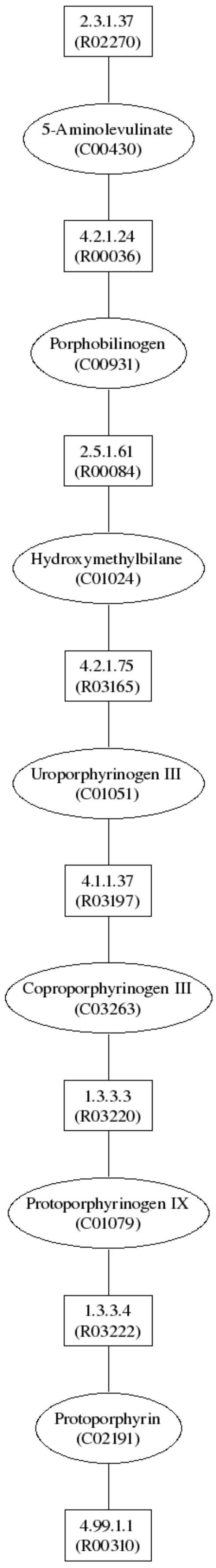

S21 Fig. Heme Biosynthesis-Yeast-C00430 C02191

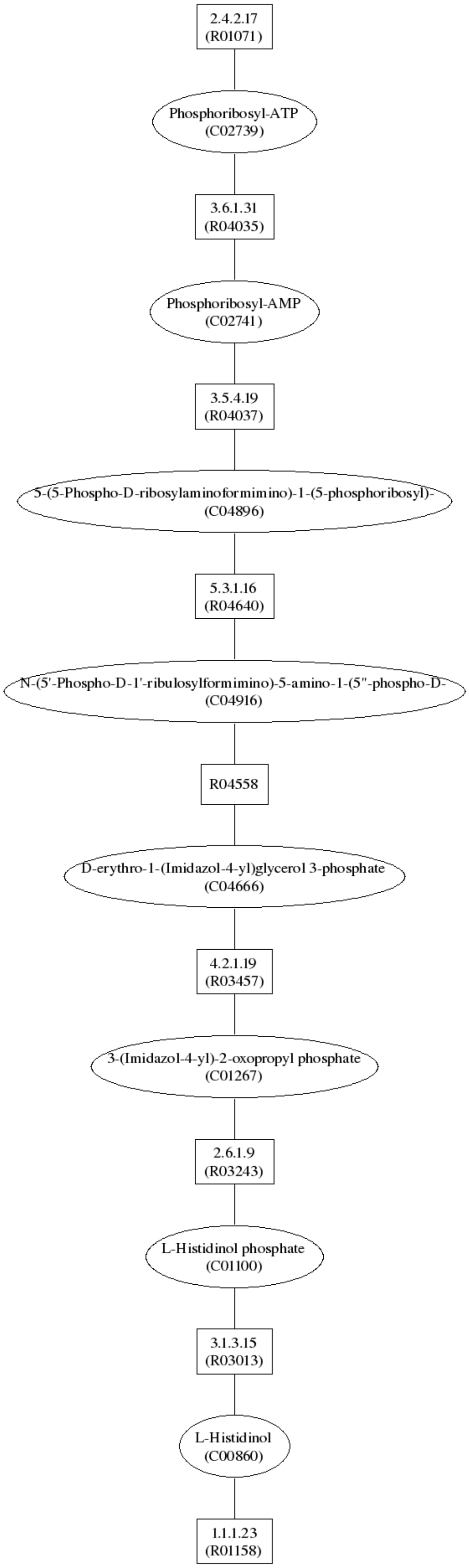

S22 Fig. Histidine Biosynthesis-Yeast-C02739 C00860

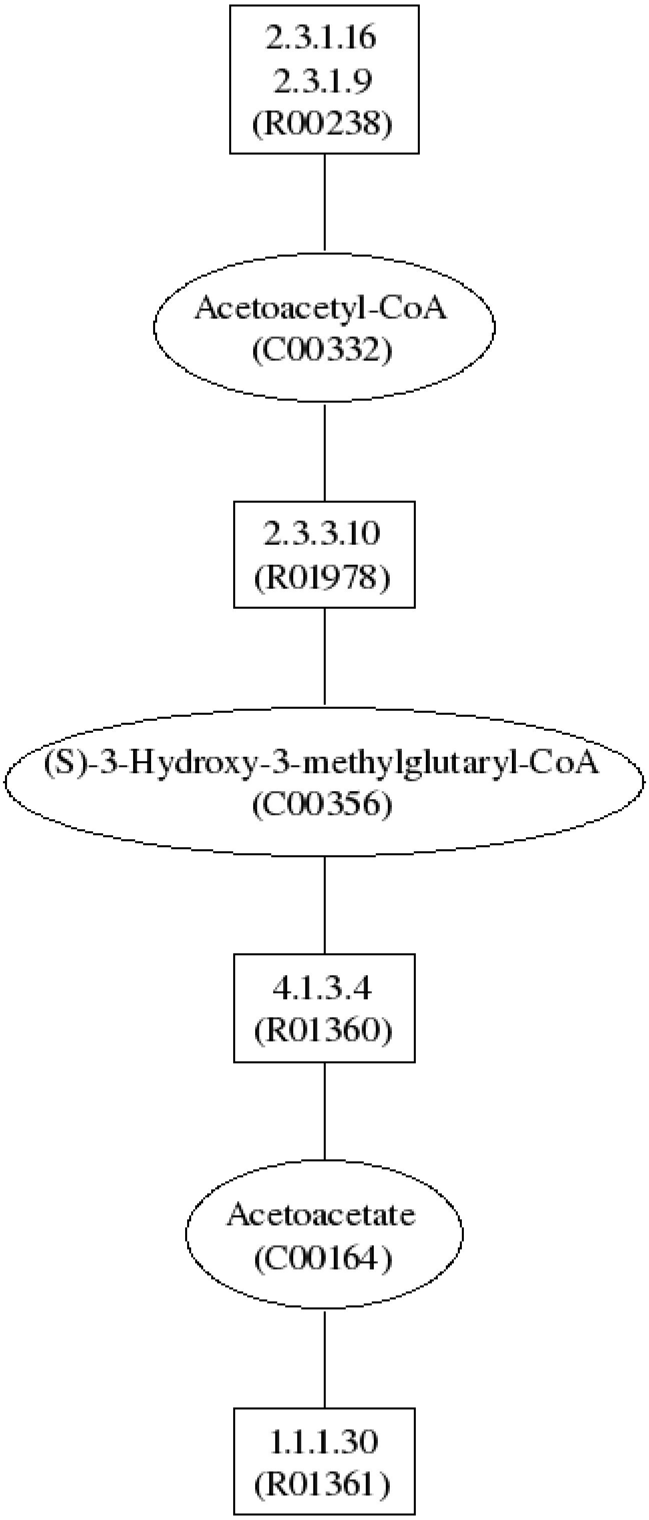

S23 Fig. Ketone Synthesis and Degradation-Human-C00332 C00164

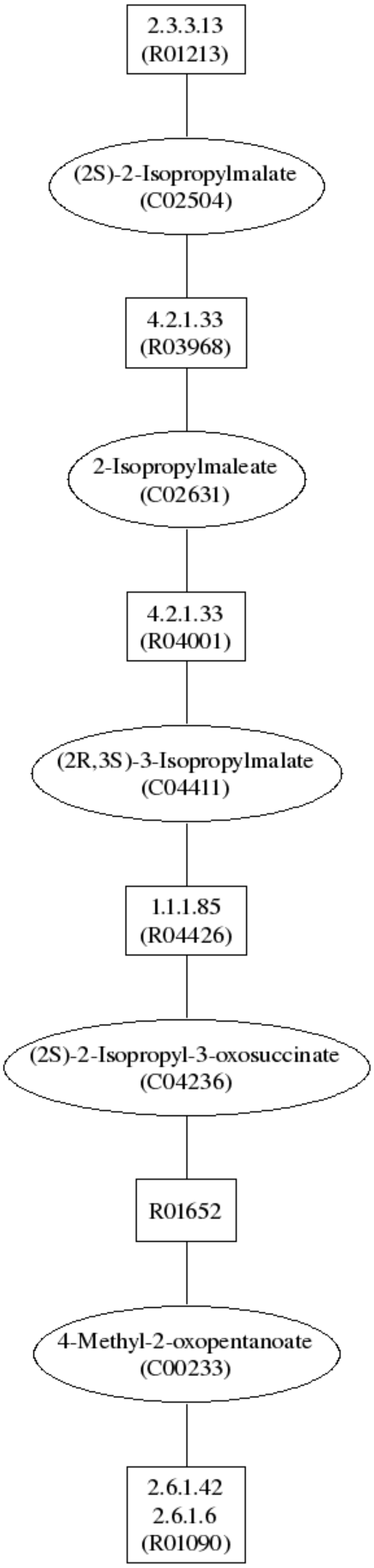

S24 Fig. Leucine Biosynthesis-Yeast-C02504 C00233

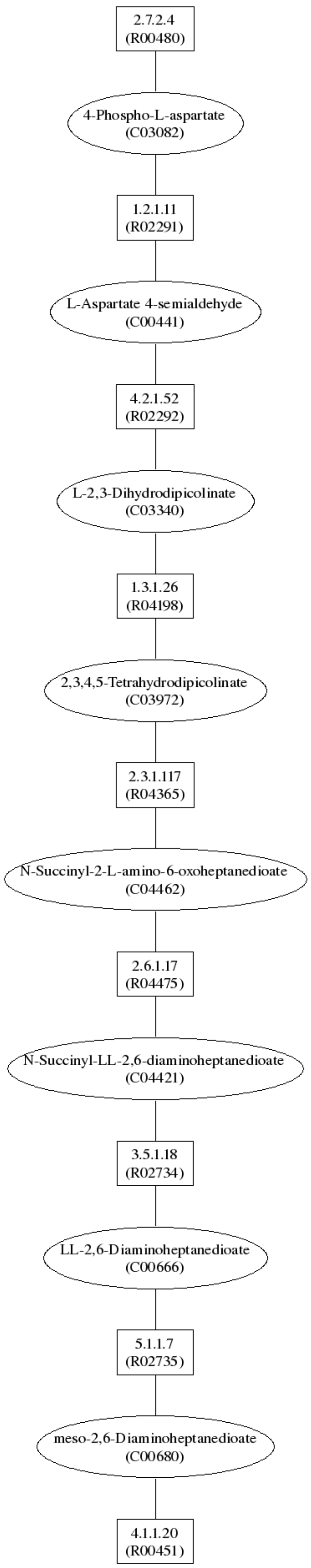

S25 Fig. Lysine Biosynthesis-Ecoli-C03082 C00680

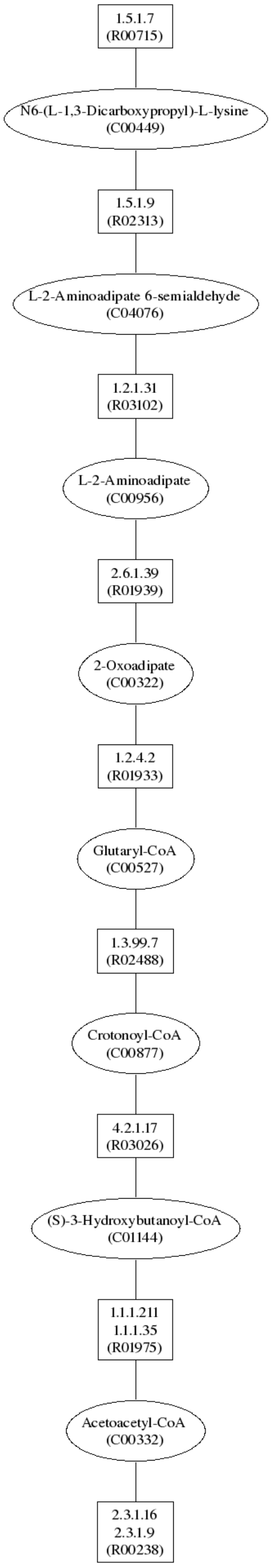

S26 Fig. Lysine Degradation-Human-C00449 C00332

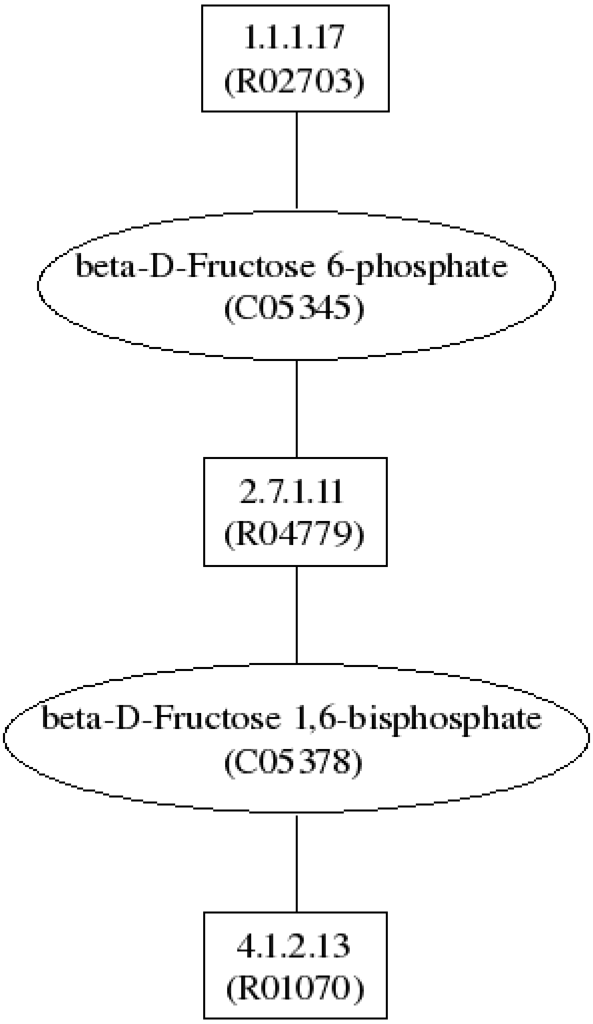

S27 Fig. Mannitol Degradation-Ecoli-C05345 C05378

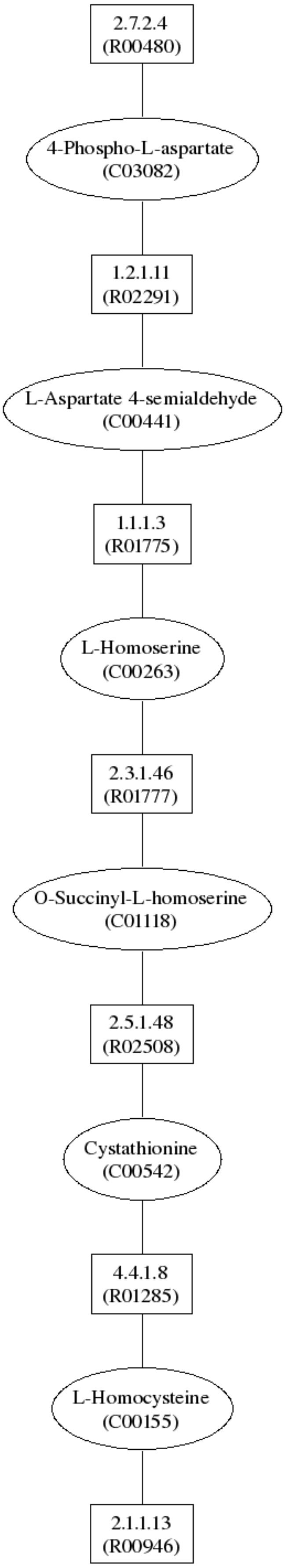

S28 Fig. Methionine Biosynthesis-Ecoli-C03082 C00155

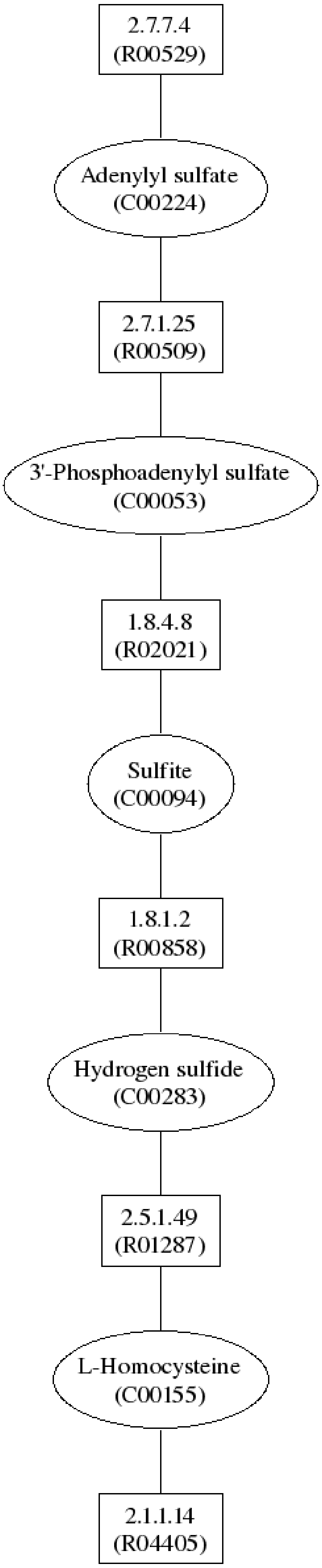

S29 Fig. Methionine BiosynthesisI-Yeast-C00224 C00155

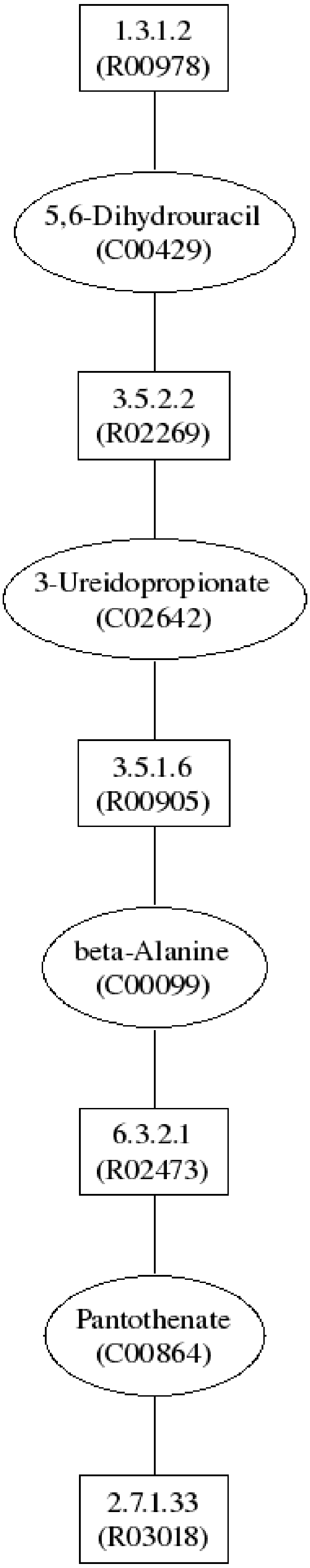

S30 Fig. Pantothenate Metabolism-Human-C00429 C00864

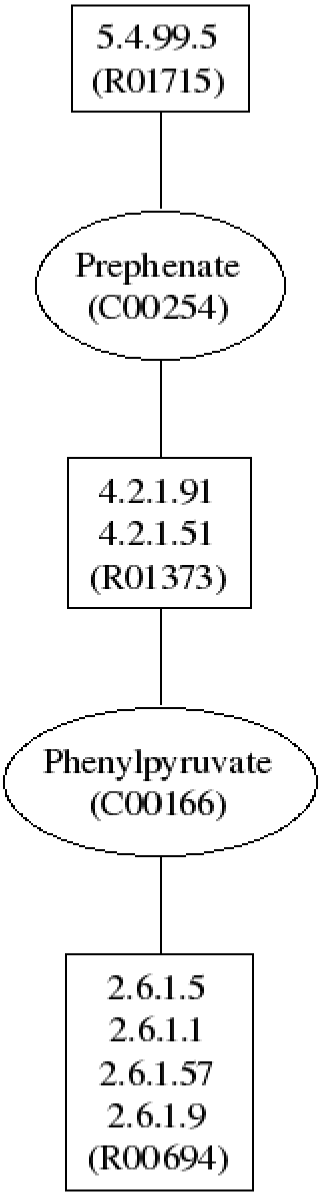

S31 Fig. Phenylalanine Biosynthesis-Ecoli-C00254 C00166

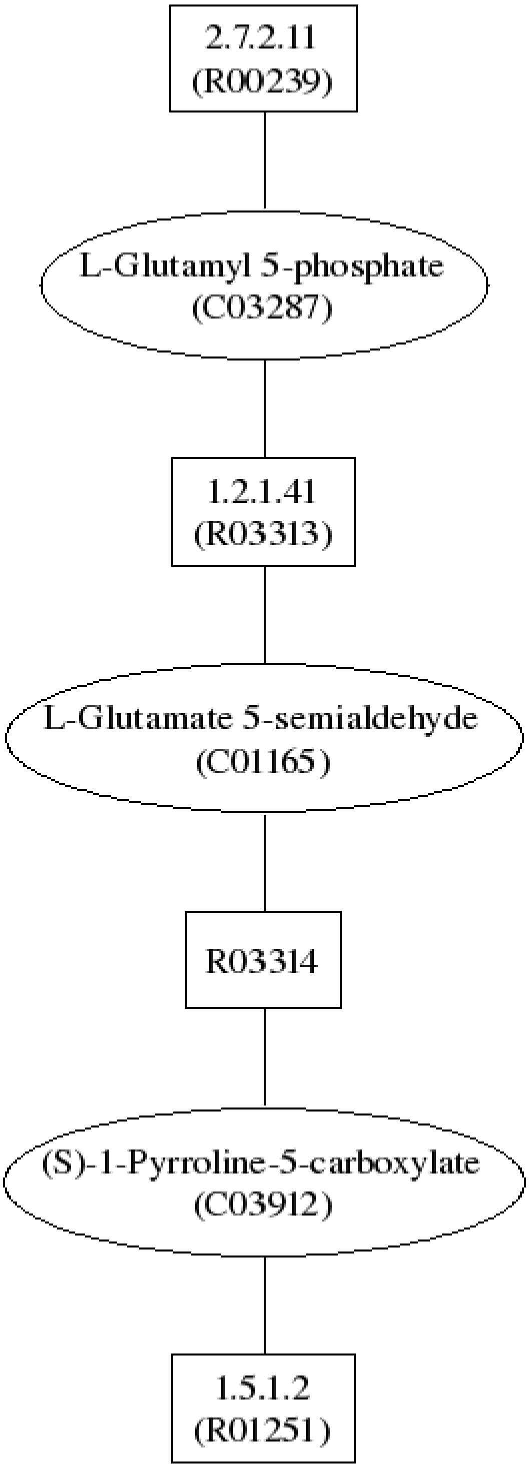

S32 Fig. Prolinebio synthesis-Ecoli-C03287 C03912

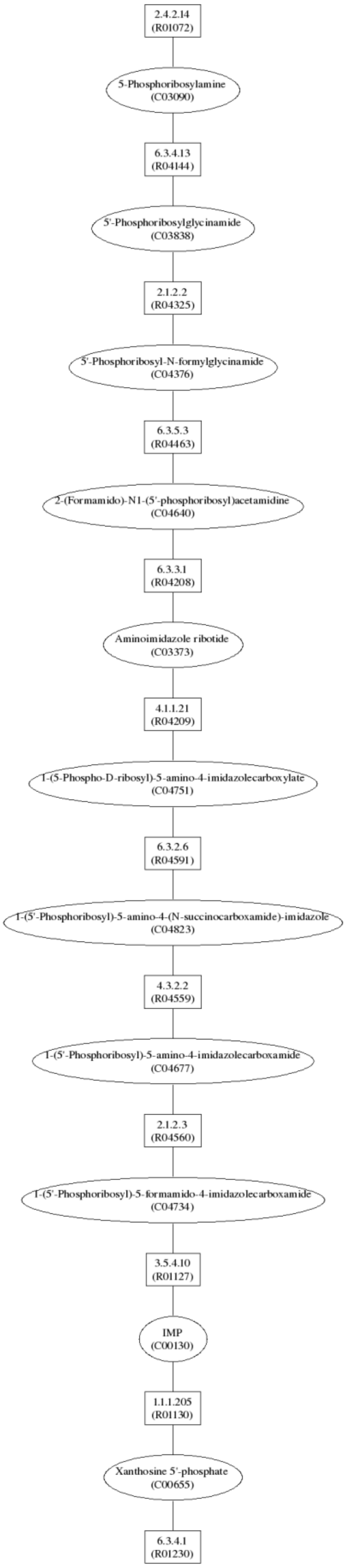

S33 Fig. Purinedenovo biosynthesis-Yeast-C03090 C00655

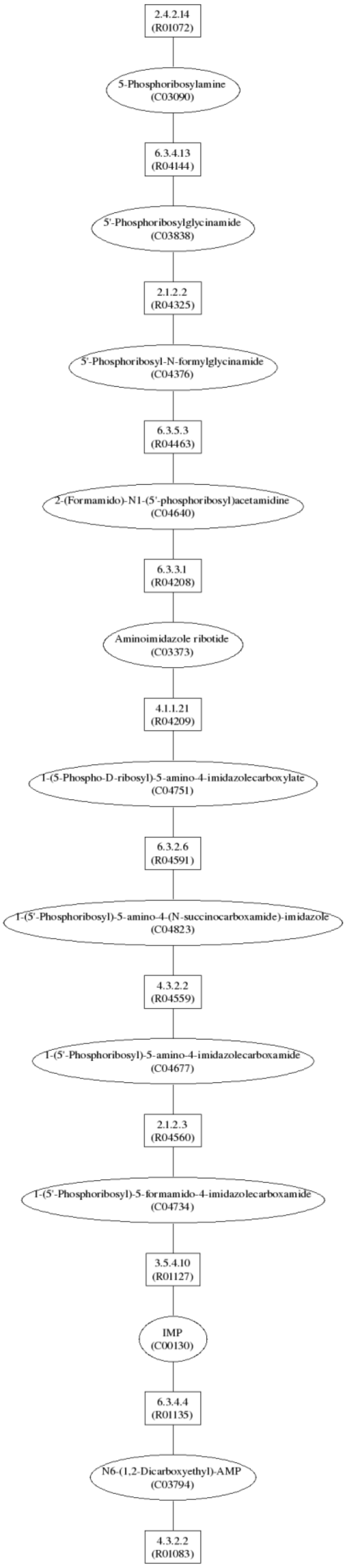

S34 Fig. Purinedenovo biosynthesis-Yeast-C03090 C03794

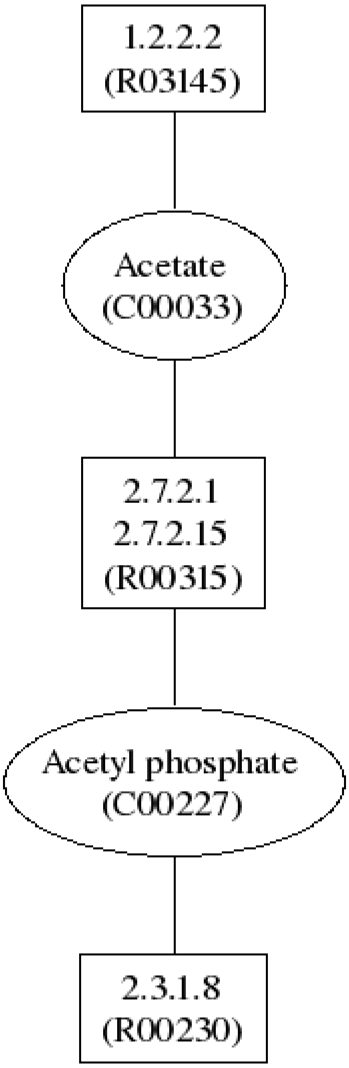

S35 Fig. Pyruvateoxidationpathway-Ecoli-C00033 C00227

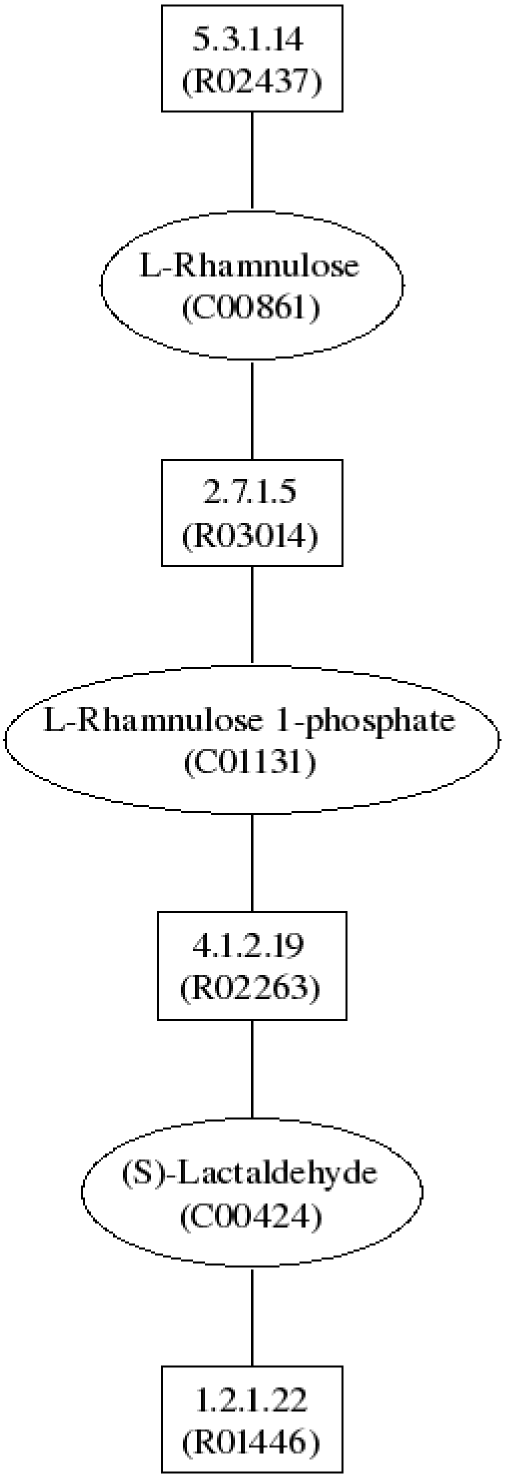

S36 Fig. Rhamnose Catabolism-Ecoli-C00861 C00424

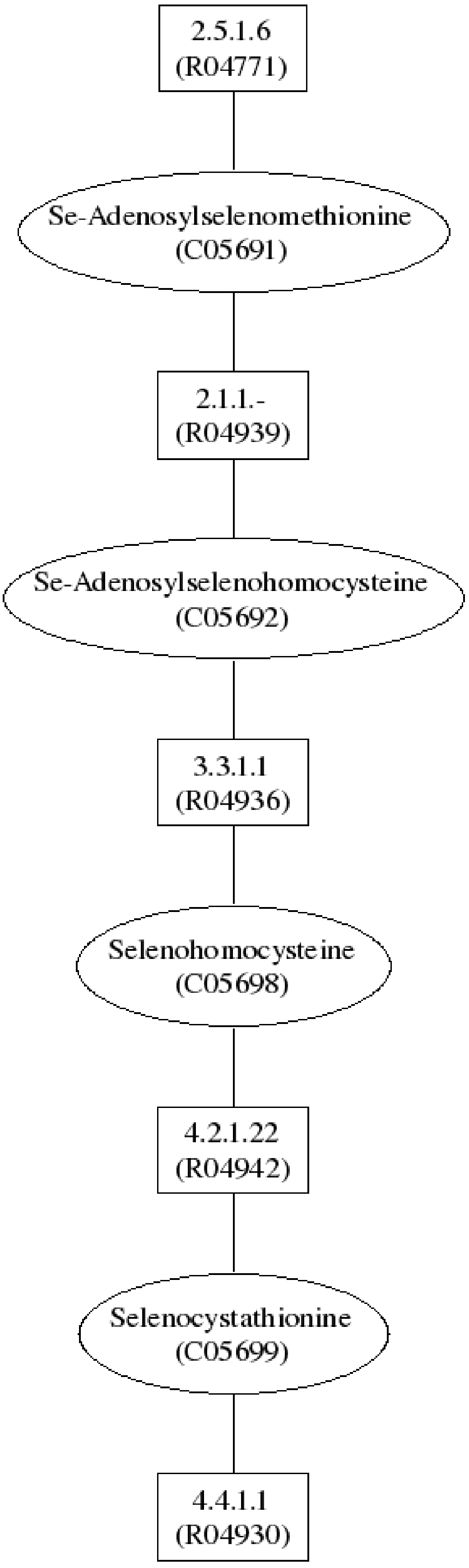

S37 Fig. Selenocysteine Biosynthesis-Human-C05691 C05699

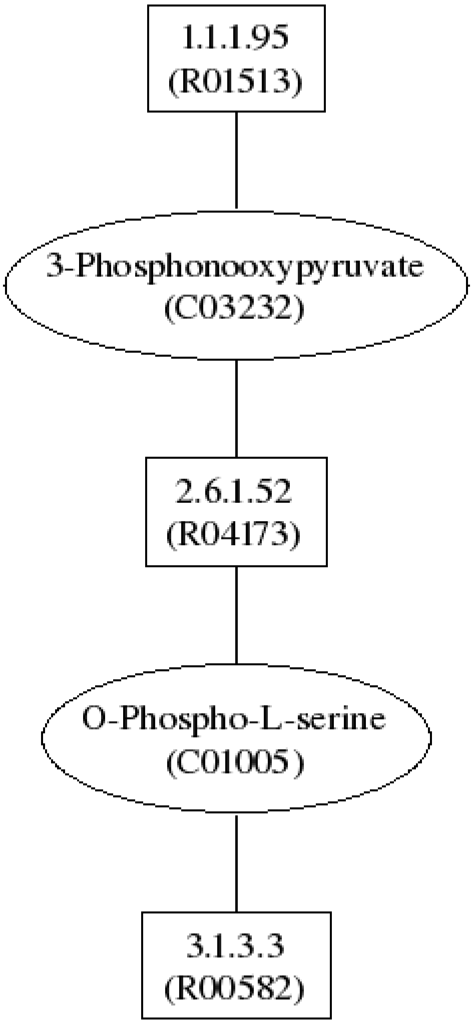

S38 Fig. Serine biosynthesis-Ecoli-C03232 C01005

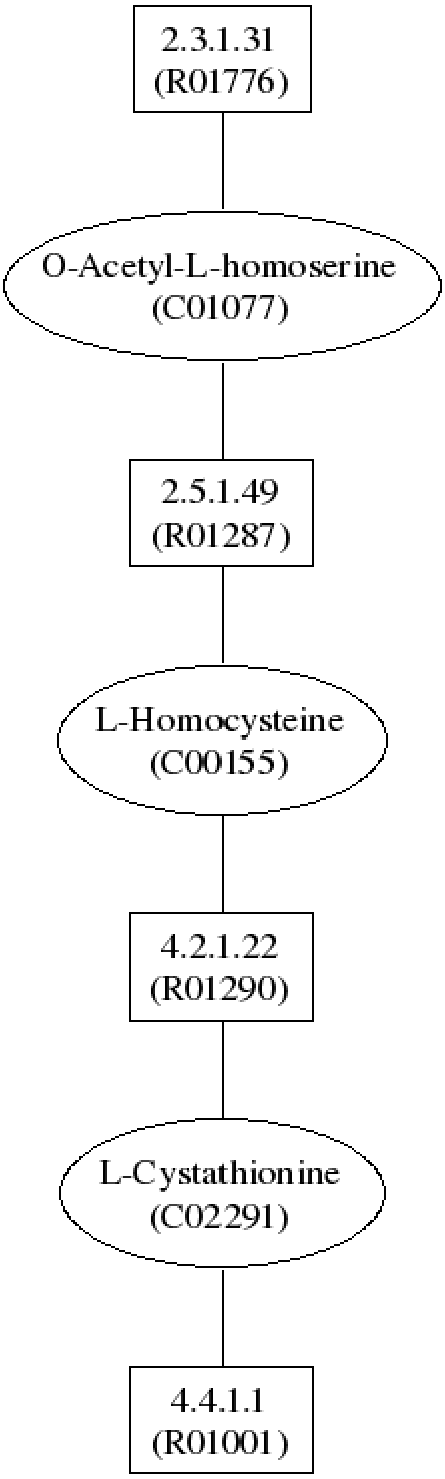

S39 Fig. Sulfur Incorporation and Transsulfuration-Yeast-C01077 C02291

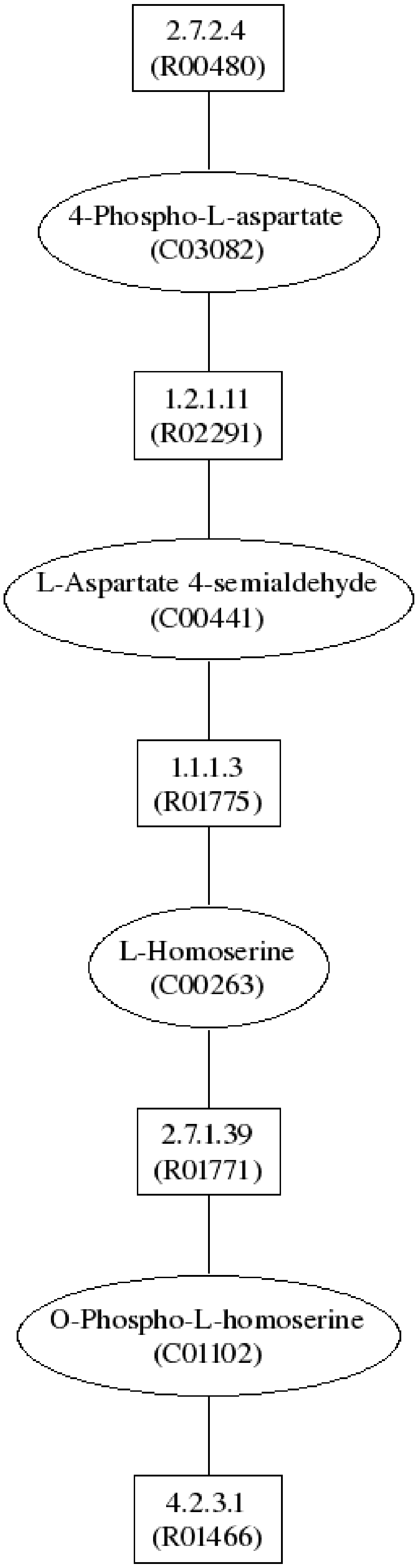

S40 Fig. Threonine Biosynthesis-Ecoli-C03082 C01102

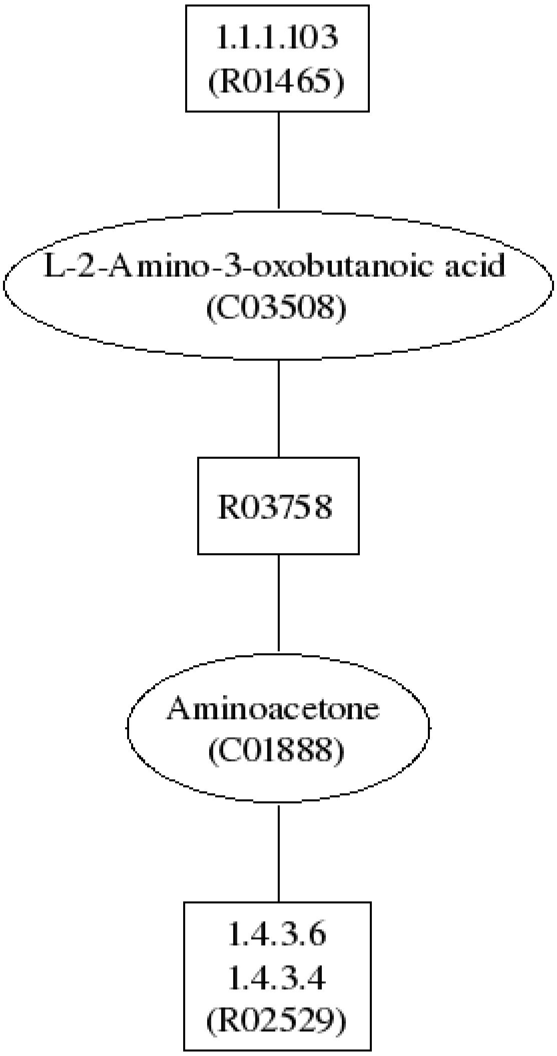

S41 Fig. Threonine Degradation-Ecoli-C03508 C01888

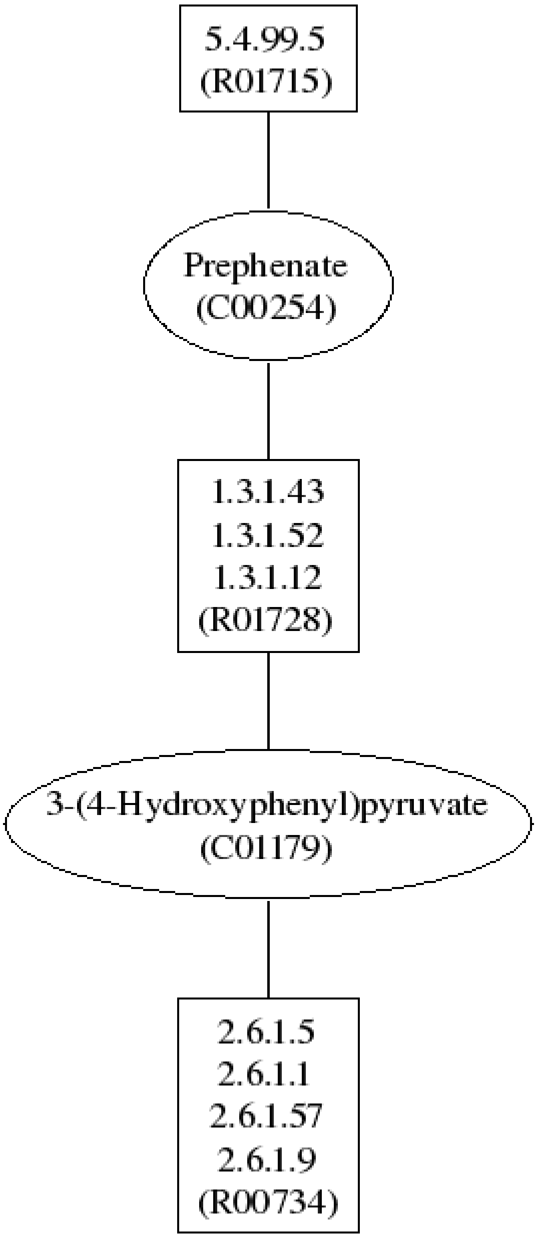

S42 Fig. Tyrosine Biosynthesis-Ecoli-C00254 C01179
