# Supplementary material for: A Method for Finding Metabolic Pathways Using Atomic Group Tracking
Source: PLoS One. 2017 Jan 9;12(1):e0168725. doi: 10.1371/journal.pone.0168725 (PMC5221824; doi:10.1371/journal.pone.0168725)
Supplement: S1 Table — (DOC) [file pone.0168725.s002.doc]

**S1 Table. The hub metabolites listed in Tinker.**

| Compound | KEGG ID |
| --- | --- |
| H_2_O | C00001 |
| H^+^ | C00080 |
| NADH | C00004 |
| CO_2_ | C00011 |
| Phosphate | C00009 |
| ADP | C00008 |
| NADPH | C00005 |
| NADP^+^ | C00006 |
| NAD^+^ | C00003 |
| ATP | C00002 |
| AMP | C00020 |
| O_2_ | C00007 |
| HP_2_O_7_^(3-)^ | C00013 |
| CoA | C00010 |
| NH_4_^+^ | C01342 |
| S-adenosyl-L-methionine | C00019 |
| GTP | C00044 |
| GDP | C00035 |
| UDP | C00015 |
| dATP | C00131 |
